# Alix-mediated assembly of the actomyosin–tight junction polarity complex preserves epithelial polarity and epithelial barrier

**DOI:** 10.1038/ncomms11876

**Published:** 2016-06-23

**Authors:** Yvan Campos, Xiaohui Qiu, Elida Gomero, Randall Wakefield, Linda Horner, Wojciech Brutkowski, Young-Goo Han, David Solecki, Sharon Frase, Antonella Bongiovanni, Alessandra d'Azzo

**Affiliations:** 1 Department of Genetics, St Jude Children's Research Hospital, 262 Danny Thomas Place, Memphis, Tennessee 38105, USA; 2 Cellular Imaging Shared Resource, St Jude Children's Research Hospital, 262 Danny Thomas Place, Memphis, Tennessee 38105, USA; 3 Laboratory of Imaging Tissue Structure and Function, Nencki Institute of Experimental Biology, Polish Academy of Sciences, 02-093 Warsaw, Poland; 4 Department of Developmental Neurobiology, St Jude Children's Research Hospital, 262 Danny Thomas Place, Memphis, Tennessee 38105, USA; 5 Institute of Biomedicine and Molecular Immunology, National Research Council, 90146 Palermo, Italy

## Abstract

Maintenance of epithelial cell polarity and epithelial barrier relies on the spatial organization of the actin cytoskeleton and proper positioning/assembly of intercellular junctions. However, how these processes are regulated is poorly understood. Here we reveal a key role for the multifunctional protein Alix in both processes. In a knockout mouse model of Alix, we identified overt structural changes in the epithelium of the choroid plexus and in the ependyma, such as asymmetrical cell shape and size, misplacement and abnormal beating of cilia, blebbing of the microvilli. These defects culminate in excessive cell extrusion, enlargement of the lateral ventricles and hydrocephalus. Mechanistically, we find that by interacting with F-actin, the Par complex and ZO-1, Alix ensures the formation and maintenance of the apically restricted actomyosin–tight junction complex. We propose that in this capacity Alix plays a role in the establishment of apical–basal polarity and in the maintenance of the epithelial barrier.

## Abstract (web summary)

Correct assembly of intercellular junctions is required to maintain epithelial polarity and barrier function. Here Campos *et al*. show that the scaffold protein Alix interacts with F-actin, the Par complex and ZO-1 to ensure the formation and maintenance of the actomyosin tight junction complex in choroid plexus epithelium.

## Graphical abstract

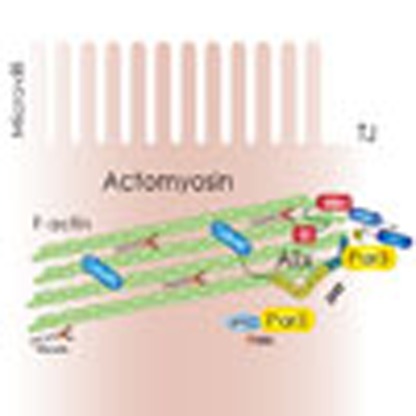

The multi-domain scaffold protein Alix (also known as PDCD6IP) was first identified as a binding partner of the proapoptotic Ca^2+^-binding protein ALG-2 (refs 1, 2). Alix has since been implicated in numerous cellular pathways because of its capacity to interact both with the lipid lysobisphosphatidic acid and with members of multiprotein complexes located at different subcellular sites^3^,^4^,^5^,^6^,^7^,^8^,^9^,^10^,^11^,^12^. Such complexes are individually entrusted to exert such fundamental functions as endocytosis, multivesicular body biogenesis, membrane repair, cytokinesis and apoptosis^13^,^14^,^15^. However, the role played by Alix in these processes has not been fully elucidated because of lack of a suitable *in vivo* model system. We have identified Alix as a substrate of the RING-type ubiquitin ligase complex Ozz-E3, which is expressed exclusively in striated muscle^16^,^17^. Silencing Alix expression in C2C12 muscle cells affects the levels and distribution of F-actin, the formation of membrane protrusions and the biogenesis of extracellular vesicles from the plasma membrane, suggesting a functional role of Alix in both membrane and cytoskeleton remodelling in skeletal muscle^3^,^18^. Adding to the complexity of Alix' functions, a proteomic study designed to investigate mechanisms of disease progression in a bacterial-induced model of colitis identified Alix as one of the most downregulated proteins in the infected intestinal epithelium^19^. Similarly, exposure of human intestinal epithelial cells to staphylococcal enterotoxin B resulted in reduced expression of Alix, which the authors suggest as the cause of epithelial barrier dysfunction^20^. In this respect, it is noteworthy that several human diseases affecting intestinal, pulmonary and renal epithelia, including cancer, have been associated with disruption of the epithelial barrier^21^.

In mammalian epithelia, cells are aligned in homogeneous layers, connected side by side by tight junctions (TJ) and adherens junctions (AJ), which together make up the apical junctional complex (AJC). The precise assembly and positioning of the junctional complex are critical for establishing the apical–basal polarity and the epithelial barrier, and also provide the mechanical strength by connecting the plasma membrane of adjacent cells to their actin cytoskeleton^22^,^23^,^24^. After establishment of cell polarity, the TJ-related proteins zonula occludens-1 and 2 (ZO-1 and ZO-2), the junctional adhesion molecules-A (JAM-A) and cingulin localize to the immature junctions^25^,^26^, and subsequently other transmembrane proteins, such as claudins and occludin, are recruited. On maturation, functional TJ undergo rearrangement of their juxtaposed actin cytoskeleton, and establish a vesicular trafficking system; these two processes enable the vectorial transport functions of the TJ^27^,^28^.

To preserve the organization of the cell layer and the integrity of the barrier, epithelia go through frequent cell renewal mediated by balanced cycles of cell division and apoptosis, using a process called ‘cell extrusion'^29^. *In vitro* studies have identified Sphingosine 1-phosphate (S1P) as the signalling molecule produced by the dying cell that activates S1P_2_ receptors in surrounding neighbouring cells, which in turn, assemble an actomyosin ring around the dying cell^30^,^31^. The latter structure mediates the extrusion of the dying cell from the cell layer, a process that is regulated by the Rho GTPase pathway^31^,^32^. Because cell extrusion is essential for regulating overall cell number and for maintaining epithelial homeostasis, alterations of any steps in this pathway may result in epithelial pathologies that either disrupt barrier function or lead to hyperplasia and cancer^33^.

In the brain, epithelial cells of the choroid plexus (CP) are the primary producers of the cerebrospinal fluid (CSF) and are responsible for establishing the blood–CSF barrier^34^. CP cells display a characteristic polarity with microvilli, cilia and TJ at their apical side and AJ, gap junctions and desmosomes or basal infoldings at the basolateral side^35^,^36^. It is noteworthy that under normal conditions fully developed CP cells do not undergo cell replacement or degeneration because the proliferation rate of CP epithelium has been shown to be <0.1% of total plexus cells per day^37^,^38^. Homeostasis of the blood–CSF barrier relies on the capacity of CP cells to regulate the movement and exchange of ions, molecules, and metabolites, and to keep the production and absorption of the CSF in balance^37^,^39^,^40^,^41^,^42^. These processes require intact TJ and involve regulated vesicular trafficking, and cytoskeletal rearrangements^43^. Because the CSF is continuously produced, pathological conditions that block or affect its normal flow and/or absorption result in the progressive accumulation of the CSF within the ventricles, increasing the pressure against the brain parenchyma and causing hydrocephalus. Despite its high prevalence in the population, the molecular bases of hydrocephalus and the mechanisms regulating CSF homeostasis are still largely unknown.

In the present study, we demonstrate that Alix ablation *in vivo* results in striking defects in the assembly and positioning of the actomyosin–TJ polarity complex in the CP, which culminate in the abnormal extrusion of cells from the epithelial layer. The latter process has not yet been linked to a specific gene defect *in vivo*. The combined abnormalities of the CP epithelium and the ependyma, associated with dysfunctional primary and motile cilia, contribute to loss of the CP homeostatic control over CSF production/absorption and flow, leading to progressive hydrocephalus. Seeking a mechanism, we find that Alix is an integral component of the actomyosin and physically interacts with F-actin, the partitioning defective protein (PAR) complex and the TJ protein ZO-1, thereby restricting the localization of these proteins to the apical domain of the epithelial cells. Overall, our findings uncover a key function of Alix in fostering the specification of a junctional complex essential for maintaining epithelial cell polarity and the integrity of the epithelial barrier.

## Results

### *Alix*-null mice develop hydrocephalus

**Figure 1:**
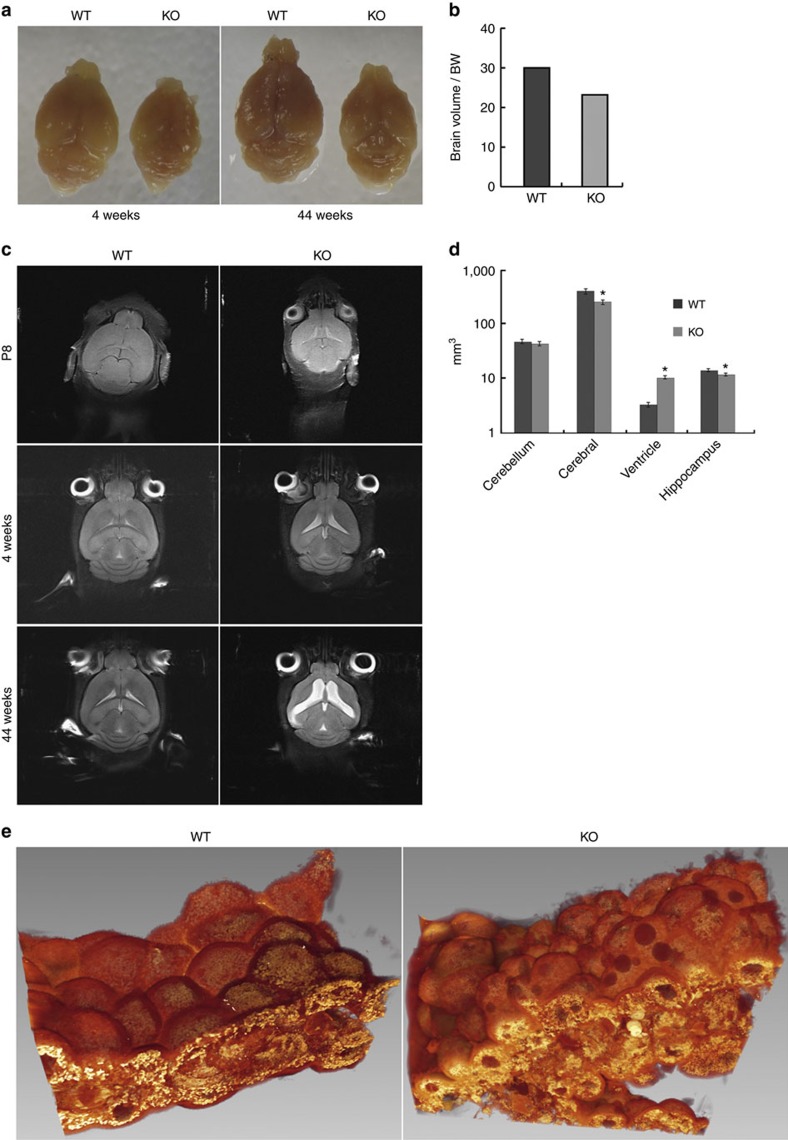
*Alix*^*−/−*^ mice develop bilateral hydrocephalus. (**a**) Brains dissected from *Alix*^*−/−*^ and WT mice (*n*=4) at different ages show overt size difference. (**b**) Quantification of the ratio between brain volume and body weight (BW) demonstrate that *Alix*^*−/−*^ brains (*n*=4) from 1-month-old mice are smaller than WT brains from mice of the same age (*n*=4). (**c**) MRI of brains from WT (left) and *Alix*^*−/−*^ (right) mice obtained starting at postnatal day 8 until 44 weeks of age. (**d**) Quantification of the volume of cerebellum, cortex, ventricles and hippocampus 1-month-old mice WT (*n*=4) and *Alix*^*−/−*^ mice (*n*=4). Data are shown as mean values±s.d. (**e**) 3D reconstruction of whole-mount scanning electron microscopy (SEM) images of the CP from the lateral ventricles.

*Alix*^*−/−*^ mice were viable and had a normal lifespan, although they were smaller in size and often identifiable by the domed shape of their head, suggestive of hydrocephalus. Histopathologically, their systemic organs looked normal, but the brain was smaller than that of wild-type (WT) mice, while the cerebellum maintained normal size and morphology (Fig. 1a,b). Sequential magnetic resonance imaging (MRI) scans of mutant and WT crania starting at postnatal day 8 until adult age revealed the presence of bilateral hydrocephalus in the *Alix*^*−/−*^ brain (Fig. 1c). This fully penetrant phenotype was associated with progressive build-up of CSF and enlargement of the lateral ventricles, thinning of the cerebral cortex and reduced size of the hippocampus (Fig. 1c,d). Ventricular enlargement, which was restricted to the lateral ventricles, became visible only after the first postnatal week (P8) not earlier, and worsened with age; there were no signs of obstruction of the Sylvius aqueduct or expansion of the fourth ventricle. Consistent with the occurrence of hydrocephalus, there was increased expression of glial fibrillary acidic protein (GFAP). The levels of GFAP were particularly high in the ependymal/sub-ependymal layer of the lateral wall, suggesting that the increased ventricular pressure caused by accumulation of CSF damaged this structure (Supplementary Fig. 1a,b).

### CP epithelium and ependyma defects in *Alix*-null mice

**Figure 2:**
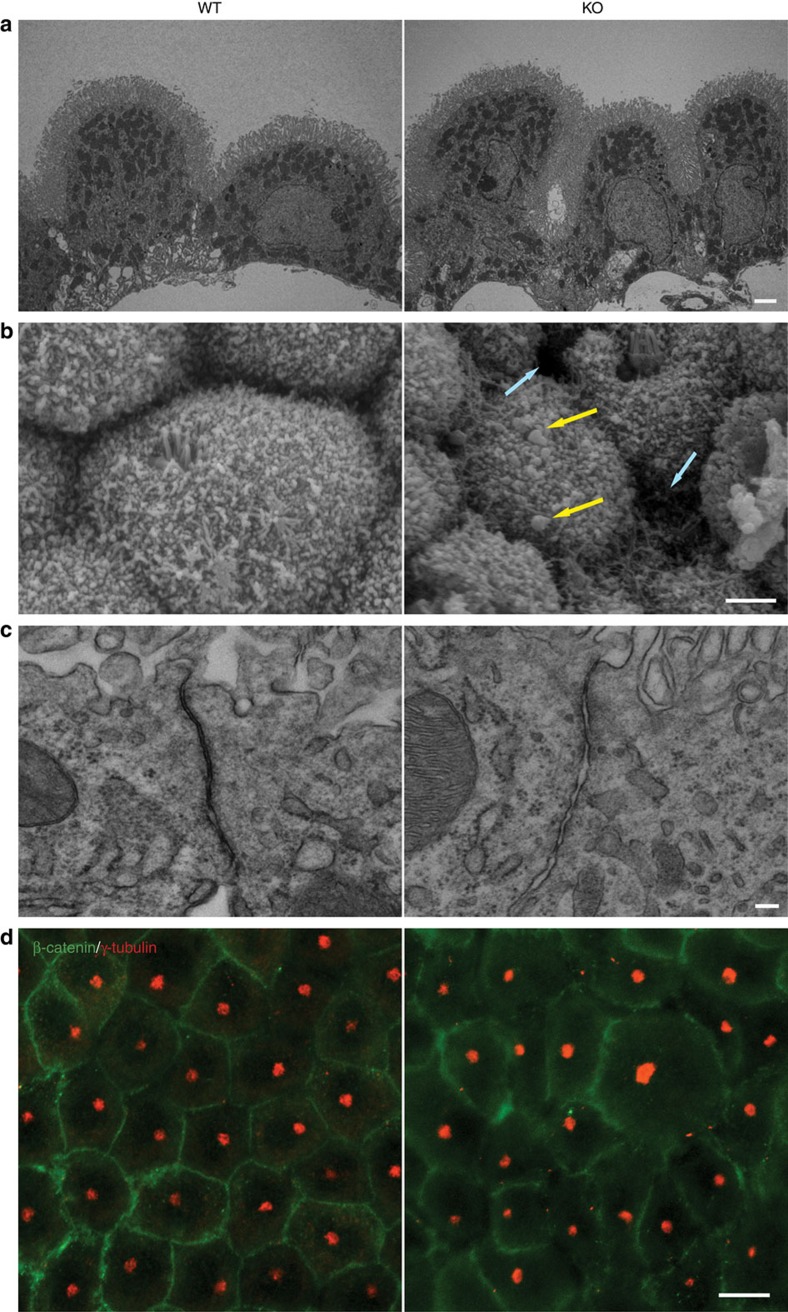
Abnormal cell architecture and AJC in the *Alix*^*−/−*^ CP. (**a**) transmission electron microscopy (TEM) revealed the misalignment of the epithelial cells, their irregular shape and size and the prominent blebbing of their microvilli (*n*=2), Scale bar, 2 μm. (**b**) SEM of the *Alix*^*−/−*^ CP apical surface showed extensive blebbing (yellow arrows) and large gaps between cells (light blue arrows) (*n*=2), Scale bar, 2 μm. (**c**) High-magnification TEM abnormally distended AJC in *Alix*^*−/−*^ CP (*n*=3), Scale bar, 100 nm. (**d**) Immunostaining of whole-mount preparations for β-catenin (green), and γ–tubulin (red) demonstrated the abnormal cell shape and mislocalization of the basal bodies in the *Alix*^*−/−*^ CP (*n*=2). Scale bar, 10 μm.

To begin dissecting the causes of the hydrocephalus and link them to Alix deficiency, we first focused on the CP epithelium, the main site of CSF biogenesis and also the highest Alix-expressing structure in the brain (Supplementary Fig. 2a,b). By three-dimensional (3D) reconstruction of whole-mount scanning electron microscopy (SEM) images, we built a holistic representation of WT and *Alix*^*−/−*^ CP that revealed overt morphological defects in the mutant CP (Fig. 1e; Supplementary Movies 1 and 2). *Alix*^*−/−*^ epithelial cell layer was not organized in one plane, as the WT CP epithelium; rather, it had an undulated appearance with vast membrane protrusions visible on the apical site (Fig. 1e; Supplementary Movies 1 and 2). Ultrastructural analysis of cross-sections of the *Alix*^*−/−*^ CP by transmission electron microscopy (TEM) highlighted the misalignment of the epithelial cells, their irregular shape and size and the prominent blebbing of their microvilli (Fig. 2a; Supplementary Fig. 3a). The latter feature was clearly distinguishable in SEM images of the CP apical surface (Fig. 2b, arrows) that also revealed the presence of large gaps between cells. Furthermore, high-magnification TEM images of the *Alix*^*−/−*^ CP epithelial cells revealed an AJC abnormally distended compared with the one in the WT cells (Fig. 2c).

The irregular morphology of the mutant epithelium was also evident by β-catenin immunofluorescence that delineated the cell contours (Fig. 2d). In addition, whole-mount immunostaining for CD9, a marker of microvilli, showed uneven expression pattern of this protein with areas devoid of staining, adjacent to others strongly positive, and further highlighted the aberrant spatial organization of the *Alix*^*−/−*^ epithelial layer (Supplementary Fig. 3b; Supplementary Movies 3 and 4). Last, γ-tubulin immunofluorescence of *Alix*^*−/−*^ CP identified ciliary basal bodies of variable size and not correctly polarized to the apical site, compared with those in the WT cells (Fig. 2d; Supplementary Fig. 3c, arrows). Thus, Alix ablation appears to ensue striking morphological defects of the CP epithelial layer, likely because of altered membrane topology and/or defective AJC.

**Figure 3:**
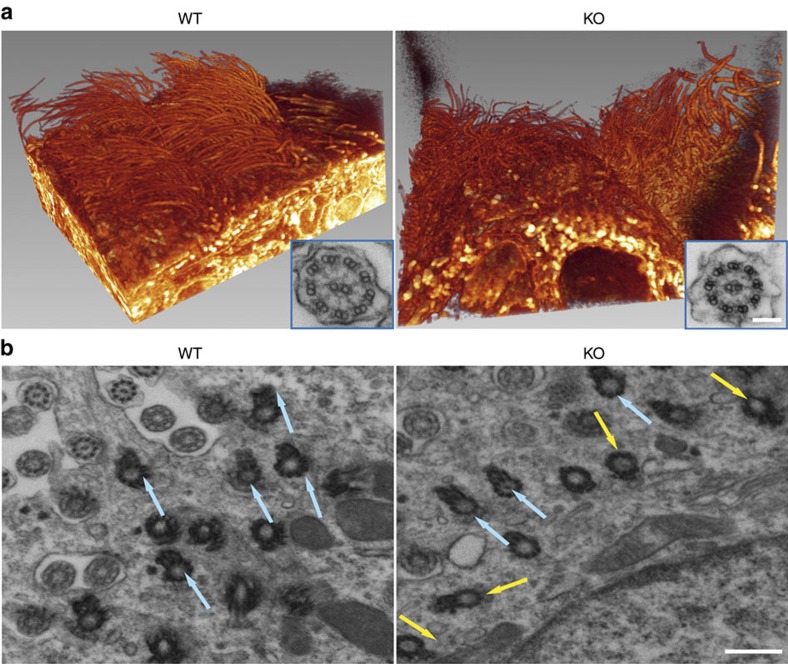
Motile cilia of lateral wall in *Alix*^*−/−*^ brain show defective orientation. (**a**) 3D reconstruction of whole-mount scanning electron microscopy (SEM) images of WT (*n*=3) and *Alix*^*−/−*^ (*n*=3) lateral wall. Inset: the typical 9+2 axoneme structure is maintained in *Alix*^*−/−*^ cilia. Scale bar, 100 μm. (**b**) Transmission electron microscopy (TEM) of the basal bodies in WT and *Alix*^*−/−*^ ependymal cells (*n*=2). Arrows indicate orientation of the basal bodies. Scale bar, 500 nm.

We next examined the ciliated ependyma of the lateral wall because we reasoned that structural alterations of the ependymal layer or malfunctioning of the motile cilia would also contribute to the *Alix*^*−/−*^ hydrocephalus. Three-dimensional reconstruction of SEM images of the *Alix*^*−/−*^ lateral wall showed altered orientation and organization of the cilia (Fig. 3a; Supplementary Movies 5 and 6), which nonetheless maintained the typical 9+2 axoneme structure (Fig. 3a, insets). Similar to the CP epithelium, whole-mount immunostaining for γ-tubulin and β-catenin revealed that in the *Alix*^*−/−*^ ependyma the basal bodies were disorderly positioned compared with those in the WT cells. In addition, individual cells were of irregular size and shape (Supplementary Fig. 4). Abnormal positioning of the basal bodies and basal feet is known to affect the orientation of the cilia, thereby impairing the coordinated beating of these organelles and the correct flow of the CSF^44^. Ultrastructural examination of the basal feet in cross-sections of the lateral wall revealed that these structures were similarly orientated in the WT ependyma, but had different orientations in the *Alix*^*−/−*^ cells (Fig. 3b). The latter defect altered the directionality of the axonemes, which resulted in asynchronous beating of the cilia (Supplementary Movies 7 and 8), and loss of CSF vectorial flow (Supplementary Movies 9 and 10). These features likely contribute to the stagnation and gradual build-up of the CSF within the lateral ventricles. Altogether, these results suggest that Alix is critical for maintaining the proper architecture and membrane integrity of both the CP epithelium and the ependymal layer lining the lateral ventricles.

### Alix ablation causes cell extrusion and loss of epithelial barrier

**Figure 4:**
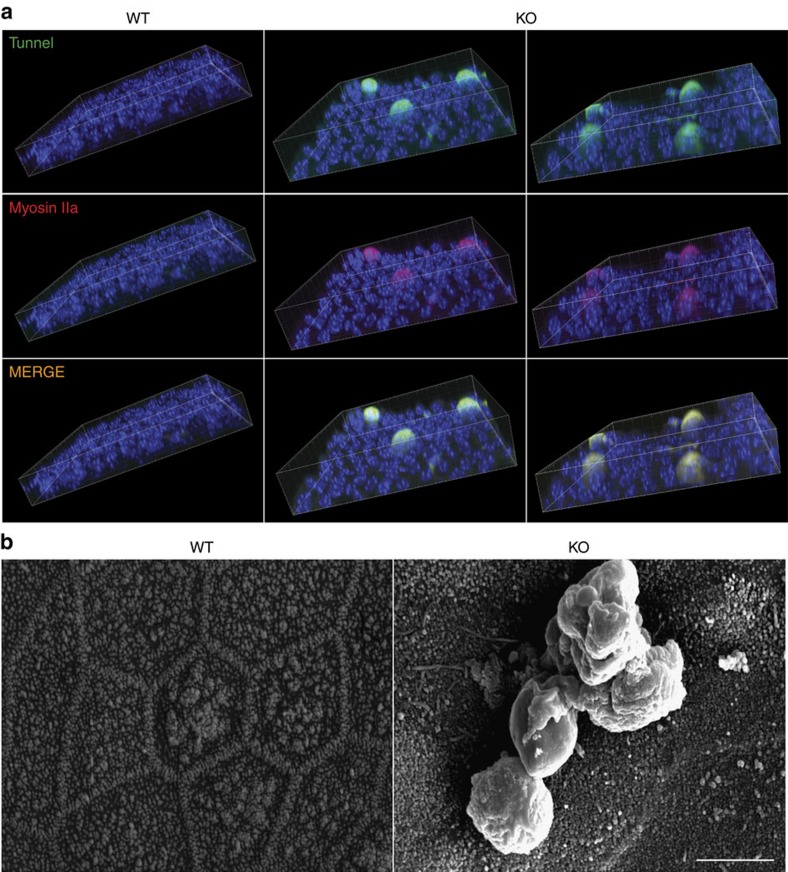
*Alix*^*−/−*^ epithelia display apoptotic cell extrusion. (**a**) 3D confocal reconstruction of WT and *Alix*^*−/−*^ whole-mount CP showing tunnel^+^ and Myosin IIa^+^ cells being extruded from epithelial cell layer of *Alix*^*−/−*^ CP (*n*=3). (**b**) SEM images of cluster of cells extruded from *Alix*^*−/−*^ tracheal epithelial cells. Scale bar, 5 μm.

An unexpected and unusual feature of the mutant CP epithelium was the presence of cells caught in the act of being extruded at the apical site of the cell layer (Supplementary Fig. 5a). To ascertain the occurrence of *in vivo* cell extrusion in the *Alix*^*−/−*^ epithelium, we performed whole-mount tunnel assays. Three-dimensional reconstruction of serial confocal images showed numerous tunnel^+^ apoptotic cells extruded from the apical site of the *Alix*^*−/−*^ epithelial layer; in contrast, no such cells were detected in the WT CP (Fig. 4a). In addition, the apoptotic cells were highly positive for the motor myosin IIa, indicating that they were undergoing extrusion (Fig. 4a). To prove that this unique phenotype was not limited to the CP, we analysed by SEM primary cultures of *Alix*^*−/−*^ tracheal epithelial cells and demonstrated that several of these cells were also apically extruded (Fig. 4b). Likewise the *Alix*^*−/−*^ CP, the tracheal epithelium exhibited the same morphological defects, such as irregular size and shape, as determined by β-catenin immunofluorescence, and abnormal distribution of the CD9 marker (Supplementary Fig. 5b). Thus, loss of Alix results in epithelial cell extrusion that combined with alteration of the membrane topology and AJC may cause perturbation of the epithelial barrier.

**Figure 5:**
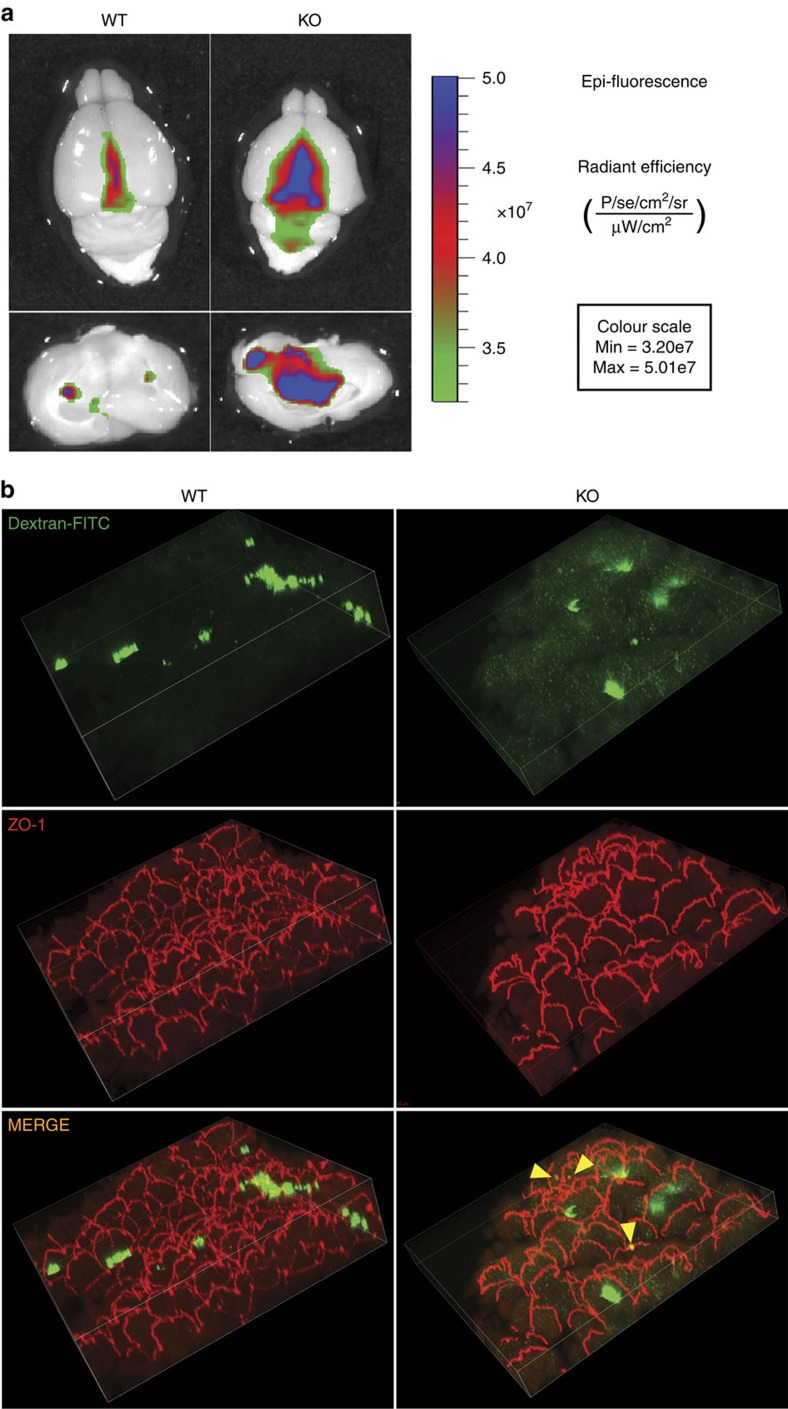
Loss of Alix compromises the epithelial barrier of the CP. (**a**) Combination of brain fluorescence and bioluminescence images obtained 24 h after intraperitoneal injection of the dye Evans blue (*n*=3). (**b**) 3D confocal analysis of WT and *Alix*^*−/−*^ whole-mount CP shows an increase intracellular flux of FITC-dextran into the *Alix*^*−/−*^ CP compared with the WT; yellow arrowheads indicate points of colocalization between FITC dextran (green) and ZO-1 (red) along the basolateral membrane of the *Alix*^*−/−*^ CP (*n*=2).

To test this hypothesis, we first used near-infrared-fluorescence analysis of WT and *Alix*^*−/−*^ brains from mice injected intraperitoneally with the dye Evans blue. Combining a photograph of the whole brain with optical images of the Evans blue fluorescence, we found that the dye was restricted to the CP in the WT brain (Fig. 5a); in sharp contrast, a large area of leakage within and around the lateral ventricles was detected in the *Alix*^*−/−*^ brain (Fig. 5a). To define the route of penetration of the dye into the CSF, we designed an *in vivo* assay, using FITC dextran injected intraperitoneally. Compared with WT CP, 3D confocal analysis of whole-mount *Alix*^*−/−*^ CP showed an increased intracellular flux of the fluorescent tracer that in some locations co-localized with ZO-1 at the TJ (Fig. 5b). In addition, we tested the flux of FITC dextran in an immortalized CP-derived cell line (Z310) (ref. 45), in which we silenced the expression of Alix. These cells were shown to maintain the structural characteristics of the CP epithelium, including proper expression and localization of TJ proteins^44^. Among the shRNA-transduced cells, Z310 *Alix* shRNA no. 2 showed about 60% knockdown of Alix expression (Supplementary Fig. 6a). These cells, cultured as monolayers in supported permeable membranes, were incubated with FITC dextran added to the medium of either the apical or basal chamber (Supplementary Fig. 6b). Quantification of the amount of fluorescence that diffused from one chamber to the other revealed a significantly increased flux of the tracer through the Alix-silenced epithelial monolayer compared with that measured in WT cells (Supplementary Fig. 6b). Furthermore, fluorescence imaging of treated cells showed an intracellular distribution of the labelled dextran in the Alix-silenced cells comparable to that observed in the *Alix*^*−/−*^ CP (Supplementary Fig. 6c). Overall these results suggest an abnormally increased transcellular and paracellular flux of the fluorescent tracer into the *Alix*^*−/−*^ CSF, likely caused by impaired epithelial barrier.

### Alix is a component of the actomyosin–tight junction complex

**Figure 6:**
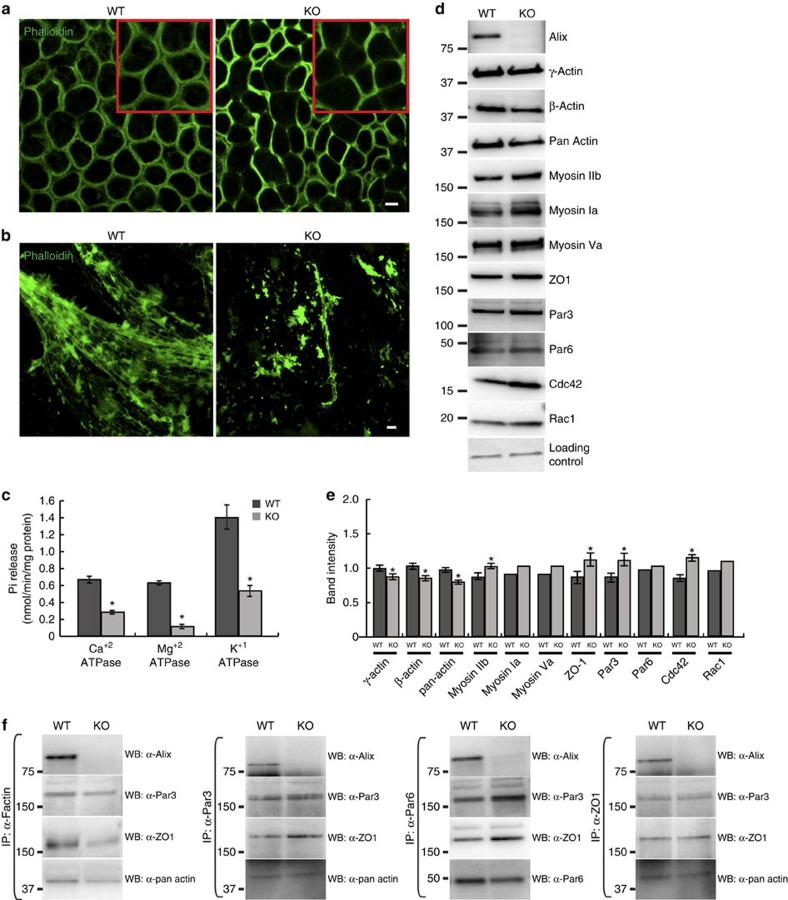
Alix maintains the integrity of actomyosin and segregates with actomyosin together with tight junction proteins. (**a**) Confocal images of whole-mount WT and *Alix*^*−/−*^ CP immunostained with phalloidin. Scale bar, 100 μm. (**b**) Purified actomyosin from WT and *Alix*^*−/−*^ brains immunostained with phalloidin. Scale bar, 10 μm. (**c**) Brain actomyosin ATPase activities, measured in the presence of Ca^2+^, Mg^2+^ and K^+^. Data are the mean±s.d. of three different actomyosin preparations. (**d**) Purified actomyosin from WT and *Alix*^*−/−*^ brains analysed by western blots for the presence of Alix and main components of actomyosin, as well as cell junctions and polarity markers. (**e**) Western blot quantification of actomyosin components. Data shown represent the mean±s.d. of three independent experiments. *P<0.05 by Student's *t*-test. (**f**) Immunoprecipitations followed by immunoblottings of subcellular fractions (P2) from WT and *Alix*^*−/−*^ brains.

Previous studies in cultured cells have revealed a role of Alix in actin cytoskeleton assembly^3^,^11^. To evaluate this function *in vivo*, we first examined the organization and composition of the actin cytoskeleton in the *Alix*^*−/−*^ CP. Whole-mount phalloidin-staining revealed a vastly uneven distribution of F-actin immunofluorescence at the cell contours of the *Alix*^*−/−*^ CP epithelium, with numerous cells showing low or absent fluorescent signal (Fig. 6a; Supplementary Movies 11 and 12).

Seeking for a molecular mechanism that would relate the morphological/barrier defects of the mutant epithelium to an altered actin cytoskeleton caused by Alix deficiency, we purified the actomyosin from WT and *Alix*^*−/−*^ brains and analysed its composition. Phalloidin immunofluorescence microscopy demonstrated that the F-actin filaments of the *Alix*^*−/−*^ actomyosin were highly fragmented and unstructured compared with those of the WT actomyosin (Fig. 6b). This resulted in defective contractile activity of the mutant actomyosin, as demonstrated by its reduced ATPase activity (Fig. 6c). Furthermore, immunoblot analysis of the purified actomyosin preparations showed that γ-actin and β-actin, the major components of F-actin, were decreased in *Alix*^*−/−*^ actomyosin, while myosin IIb, myosin Ia and myosin Va were slightly increased, denoting the abnormal composition and assembly of the actomyosin in absence of Alix (Fig. 6d,e).

A unique finding was that Alix co-purified with the assembled F-actin filaments in the WT actomyosin together with the PAR proteins Par3, and Par6, the TJ protein ZO-1, and two members of the Rho family of small GTPases, cdc42 and Rac1 (Fig. 6d,e). The levels of these proteins were distinctly higher in the *Alix*^*−/−*^ actomyosin than in the WT (Fig. 6d,e), suggesting a role of Alix in maintaining their homeostatic levels and proper positioning at the apical site of the epithelial cells. Another component of the TJ polarity complex, the integral membrane protein JAM-1, was instead not detected in the actomyosin preparations. Similarly, other TJ integral membrane proteins, that is, Occludin and Claudin-3, were also absent (Supplementary Fig. 7a). It is noteworthy that β-catenin, which associates with E-cadherin at the AJ, was also undetectable in the actomyosin preparations, a finding that confirms that only some components of the TJ polarity complex co-purify with Alix and F-actin (Fig. 6d; Supplementary Fig. 7a).

To verify the interaction of these endogenous proteins *in vivo*, the membranous/cytoskeletal subcellular fractions purified from WT and *Alix*^*−/−*^ brain lysates were immunoprecipitated with F-actin, Par3, Par6 or ZO-1 antibodies, and probed on immunoblots with Alix, Par3, ZO-1, pan-actin and Par6 antibodies. The results showed that Alix indeed co-immunoprecipitated with F-actin and with components of the polarity complex, indicating that these proteins directly or indirectly interact with each other (Fig. 6f). Western blot analysis confirmed the presence of TJ and AJ proteins in both WT and *Alix*^*−/−*^ subcellular fractions before immunoprecipitation (Supplementary Fig. 7b).

### Loss of Alix affects epithelial cell polarity

**Figure 7:**
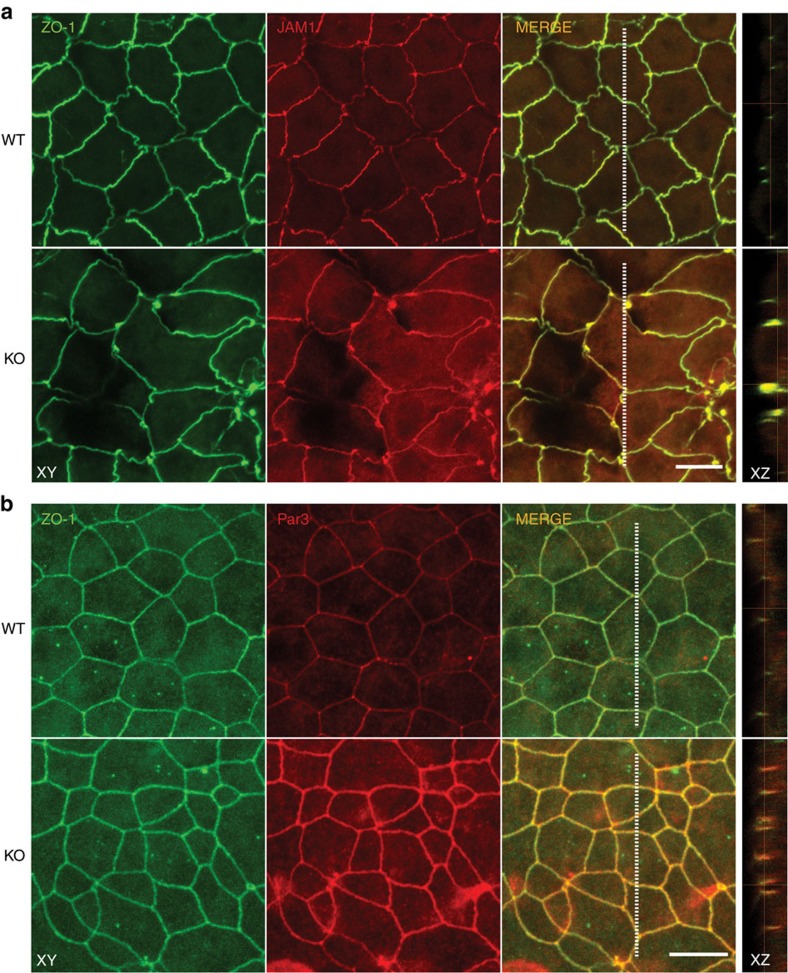
*Alix*^*−/−*^ CP shows defects in epithelial cell polarity. (**a**) Maximum intensity projections (MIP) of confocal microscopy images of whole-mount WT and *Alix*^*−/−*^ CP (*n*=3) immunostained for ZO-1 (green) and JAM-1 (red). (**b**) WT and *Alix*^*−/−*^ tracheal epithelial cell cultures immunostained for ZO-1 (green) and Par3 (red). The dotted lines in the XY images indicate the corresponding XZ sections. Data are representative of at least three independent experiments. Scale bar, 10 μm.

Given the known relationship between proper positioning and assembly of TJ proteins, and establishment of apical–basal polarity, we tested whether ZO-1 (Supplementary Movies 13 and 14) co-localized with other canonical TJ proteins, such as JAM-1, Occludin and Claudin-3, in whole-mounts of WT and *Alix*^*−/−*^ CP (Fig. 7a; Supplementary Fig. 8a,b). Although these proteins co-localized in both the samples, in the *Alix*^*−/−*^ CP there were large areas with no fluorescent signal and others with abnormal accumulation of these proteins, often in aggregates, extending toward the basal site of the cells (Fig. 7a, XY and XZ projection). Three-dimensional reconstruction of serial confocal images encompassing both epithelial layers stained for Occludin displayed the extensive disorganization of the *Alix*^*−/−*^ CP (Supplementary Movies 15 and 16). The abnormal distribution of TJ proteins in the mutant CP that extended towards the basolateral domain of the membrane implied loss of TJ integrity, which could directly affect cell polarity. To test this, we examined the localization of Par3 in WT and *Alix*^*−/−*^ tracheal epithelial cell cultures. We found that Par3 co-localized with ZO-1 in both cell cultures (Fig. 7b). However, in the mutant cells there was a striking accumulation of Par3, corroborating the results obtained with the *Alix*^*−/−*^ actomyosin preparations (Figs 7b and 6d). In addition, in the mutant cells both proteins were again aberrantly distributed throughout the apicolateral borders of the cell–cell contacts (Fig. 7b, XZ projection). These results point to a role of Alix in determining the correct localization of TJ proteins along the apical–basal axis of the epithelial cells.

### Alix is involved in TJ reassembly

**Figure 8:**
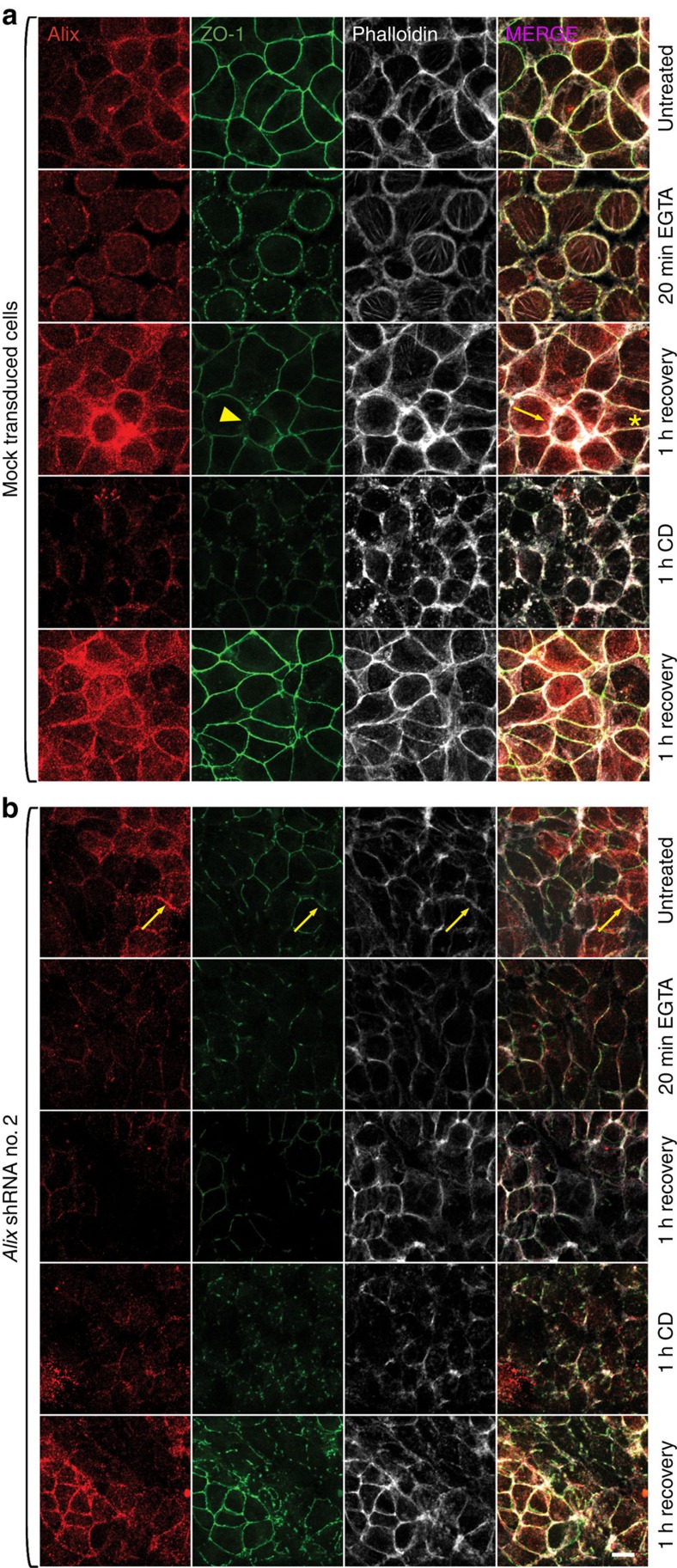
Alix is involved in TJ reassembly. (**a**,**b**) Z310 CP cell line transduced with mock or *Alix* shRNA lentiviral constructs. Cells were treated with 2 mM EGTA or 10 μM cytochalasin D (CD) and allowed to recover. Scale bar, 10 μm.

To monitor the dynamic involvement of Alix during TJ reassembly we again used the Z310 CP cell line with silenced Alix expression. Compared with mock-transduced cells, where Alix and F-actin co-localized at cell–cell contact sites, the *Alix* silenced cells showed a decreased F-actin signal with a diffused and uneven distribution (Supplementary Fig. 9), similar to that observed in the *Alix*^*−/−*^ CP epithelium (Fig. 6a). Using this *in vitro* system, we then tested the effects of Alix knockdown in the reassembly of TJ, after chemically dismantling them with EGTA, and in the ability of TJ proteins to interact with F-actin, after treatment with cytochalasin D (CD). In mock-transduced cells not treated with EGTA, ZO-1 was correctly localized at the apical side of the cell–cell contacts (Fig. 8a). After addition of EGTA, the majority of the cells rounded up and detached from their neighbours. In these cells, ZO-1 appeared fragmented but still was able to associate with both F-actin and Alix (Fig. 8a). One hour after the removal of EGTA, most cells had reassembled the TJ with their neighbouring cells. ZO-1, Alix and F-actin were properly localized wherever the TJ were fully restored (asterisk), while the areas still undergoing TJ remodelling (arrowhead) showed very high expression of Alix and F-actin (arrow; Fig. 8a). The *Alix* silenced culture contained a mixed population of cells that were either devoid of Alix or expressed very low levels of the protein; whenever present, Alix distribution and localization overlapped with those of ZO-1 and F-actin (Fig. 8b, arrows). Cells devoid of Alix had abnormal formation of TJ, while cells with residual Alix expression were still able to assemble the TJ, albeit F-actin distribution was overall weak and fragmented (Fig. 8b). These data agreed with the reduced actin-containing filaments in the *Alix*^*−/−*^ CP epithelium. Interestingly, after EGTA treatment, cells with low expression of Alix also showed a slow rate of reassembly of the TJ and were not able to fully reconstitute the TJ (Fig. 8b).

On exposure to CD, mock-transduced cells underwent extensive redistribution of F-actin filaments and formed irregular aggregates of polymerized F-actin, which also stained positive for Alix and ZO-1 (Fig. 8a). One hour after CD removal, treated cells quickly re-established polymerization of the F-actin filaments; at the same time, Alix and ZO-1 were targeted to the TJ (Fig. 8a). In cells devoid of Alix, treatment with CD did not seem to further exacerbate the F-actin depolymerization; the restoration of the actin-containing filaments and TJ remained defective during the recovery time (Fig. 8b). The striking defects in F-actin and TJ organization in the *Alix*^*−/−*^ CP epithelium and in the Alix silenced CP cells define a novel role of Alix in maintaining the proper connections between the actin cytoskeleton and the TJ proteins at the apical side of the epithelial cell membrane.

## Discussion

In polarized epithelial cells, the crosstalk between the actin-containing-filament cytoskeleton, forming the actomyosin network, and the TJ complexes, specified by ZO-1 and its interacting proteins, is essential for preserving the general architecture of the epithelium and the epithelial barrier^46^,^47^,^48^. It is also well-established that perturbation of actin dynamics affects the functions of the epithelial barrier and leads to disease^47^,^49^,^50^,^51^. However, the molecular signals that regulates the connection between different TJ complexes and the actomyosin network to support a functional epithelial barrier are still poorly understood in a mammalian system^50^,^51^. Our study has unveiled an unanticipated function of the scaffold protein Alix in establishing such link, offering a novel molecular mechanism for the maintenance of TJ integrity and, in turn, the epithelial barrier. We show that loss of Alix causes stricking defects in the actomyosin, the TJ, and their connections, leading to prominent changes in cell morphology and loss of cell polarity. These phenotypic alterations culminate in pathologic levels of epithelial cell extrusion. Physiologically, this process, which allows for the ordered removal of cells from the epithelial layer, while maintaining the integrity of the barrier^52^,^53^, is responsible for the homeostatic control of epithelial cell number during developmental tissue morphogenesis^30^,^32^,^33^. Deregulated control of epithelial cell extrusion has been linked to tumour cell invasion and metastasis^54^,^55^. However, the signals that regulate the occurrence of this process in the epithelium have been investigated predominantly using *in vitro* models. Therefore, the *Alix* knockout mice may represent a valuable model to study the molecular pathways that control epithelial cell extrusion *in vivo*.

The combined malformations of the CP epithelium and the ependyma in the *Alix* knockouts ultimately result in severe hydrocephalus. The CP, as the site of the blood–CSF barrier, is the protective entity that ensures proper CSF production and composition^37^. Alterations in the CP that affect the flow, synthesis and/or absorption of the CSF lead to net accumulation of the CSF within the ventricles and consequently to hydrocephalus^56^,^57^. In spite of its prevalence in the human population, the molecular bases of this disorder have remained largely unknown. Thus, the occurrence of progressive and severe hydrocephalus in our mouse model identifies Alix as a potential predisposing factor for this clinical condition in humans.

**Figure 9:**
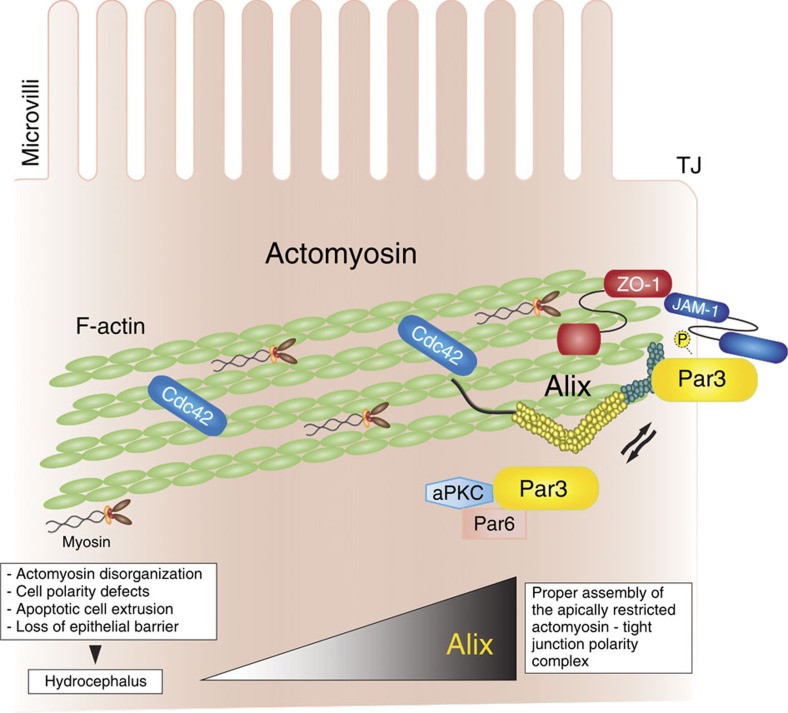
Model of the mode of action of Alix at the apically restricted actomyosin–tight junction (TJ) complex in the CP. In normal CP cells, Alix by interacting with F-actin, Par 3 and ZO-1 secures the proper assembly and positioning of an actomyosin–TJ complex at the apical sides of adjacent epithelial cells that defines a spatial membrane domain essential for the maintenance of epithelial cell polarity and barrier. Alix ablation in the CP affects the formation/maintenance of the actomyosin–TJ polarity complex with consequent loss of epithelial polarity and barrier, and progressive hydrocephalus.

In search for mechanisms that would explain this phenotype downstream of Alix deficiency, we focused on the composition of the actomyosin network and the functionality of the blood–CSF barrier. Our reasoning was dictated by the fact that the phalloidin-stained F-actin in the CP, and the brain actomyosin morphology and contractile activity were clearly defective in the *Alix*-null mice. Curiously, we noticed that a proteomic and bioinformatic analysis of epithelial TJ listed Pdcd6ip (Alix) as one of the components^58^. Our results demonstrate that in pure actomyosin preparations Alix co-purifies with the polarity complex proteins Par3 and Par6 and the TJ scaffold protein ZO-1, and that all these four proteins co-immunoprecipated with F-actin. Furthermore, our *in vitro* TJ reconstitution experiments in CP cells confirmed the co-localization of Alix, F-actin and ZO-1 only during TJ assembly, indicating that these proteins actively cooperate in this process. Altogether these intriguing observations support the notion that Alix is an integral component of the apically restricted actomyosin–TJ polarity complex. We propose that in this setting Alix fine-tunes the proper assembly of a functional actomyosin—Par complex, positioning it in close proximity to the ZO-1/JAM-1 apical membrane microdomain (Fig. 9). This configuration would promote the efficient activation of the Par complex through its interaction with the small Rho GTPases cdc42 and/or Rac1 (Fig. 9). The latter proteins have been implicated in actin polymerization and branching, TJ formation and activity of the Par complex^59^. Maintaining their homeostatic levels has proven essential for preserving the integrity of the epithelial barrier^23^. As a consequence of Alix deficiency, we observed abnormal distribution and localization of TJ proteins in the CP, and abnormal extension of the TJ towards the basolateral side of the membrane. These features lead to loss of TJ integrity, which directly affects cell polarity and blood–CSF barrier, and ultimately results in the development of hydrocephalus.

It was shown earlier that mutations in the intraflagellar transport gene Tg737 causes abnormal formation of the primary cilia of the CP and the motile cilia of the ependyma in the Tg737orpk mouse model^56^. These mice developed hydrocephalus, a phenotype that the authors attributed primarily to altered function of the CP primary cilia, which affects their ability to regulate ion transport and CSF production, leading to disorganized beating of the ependymal motile cilia and abnormal CSF flow^56^. Furthermore, altered cell polarity in the ependymal layer of the lateral ventricles is known to affect the orientation of the motile cilia, which consequently beat in an asynchronous and uncoordinated way, altering CSF flow^60^,^61^,^62^. In line with these studies, we now show that Alix deficiency causes structural defects in the orientation and organization of the cilia of the CP epithelium and the ependyma, due to altered architecture of the actin cytoskeleton. The observed defects in the cilia further contribute to the occurrence of hydrocephalus by deregulation of CSF production and flow.

In conclusion, we have identified an important role of Alix in preserving the characteristics and functionality of an apical TJ complex responsible for epithelial cell polarity. Alix exerts these functions by securing the correct positioning and interactions of junctional proteins within a macromolecular assembly with the actomyosin cytoskeleton (Fig. 9). These findings add a tier to the regulation of junctional complexes in the epithelium that extend beyond the CP and the ependyma, given that we observed identical alterations in the tracheal epithelium. It is tempting to speculate that the diverse functions of Alix may be primarily related to the capacity of this scaffold protein to directly interact with the actin cytroskeleton, thereby creating the most favourable setting for components of multiprotein complexes to assemble in a timely, spatial and dynamic manner and to target them to specific cellular microdomains. Finally, the specific defects of the actomyosin network, accompanied by loss of epithelial cell morphology/polarity and proper TJ assembly, explain the occurrence of hydrocephalus in the *Alix*-null mice and underscore the importance of fine-tuning the function of epithelial cells in the brain. More studies, especially in the clinical setting, are needed before the full magnitude and clinical relevance of our findings can be revealed.

## Methods

### Generation of the *Alix*-null mice

The murine *Alix* gene, isolated from 129sv.genomic library, was targeted by homologous recombination in W9.5 ES cells using standard procedures (Nastasi *et al*.,^17^). Disruption of the *Alix* gene was achieved by deletion of 118 bp corresponding of exon 2 and partially exon 3. ES clones were screened by PCR using a reverse primer outside the targeting construct (11,006–11,026 bp) in conjunction with two forward primers, the first located (7,732–7,750 bp) and the second (10,380–10,399 bp) downstream of the Alix start codon. Four homologous recombined ES clones with a correct karyotype were injected into C57BL/6 blastocysts. Of 129sv chimeric mice, two gave germline transmission and were crossed with both 129sv and C57BL/6 mice to generate *Alix*-heterozygous animals, which were then interbred to obtain *Alix*^*−/−*^ mice. Northern blot and western blot analyses were performed as described earlier^17^ (Supplementary Fig. 10). All mouse experiments were performed in compliance with our animal protocols approved by the St Jude Institutional Animal Care and Use Committee, and with the National Institutes of Health guidelines. In total, 275 *Alix*^*−/−*^ mice were used for this study. This number comprised all mice used for the initial molecular, biochemical and histopathological characterization of this model. Experimental procedures included histopathology, immunohistochemistry, blood and serum analyses, CSF collection and electron microscopy.

### Antibodies and reagents

Rabbit anti-Alix antibody was prepared as described^3^. The antibody was diluted 1:500 for western blotting and 1:50 for immunofluorescence. Primary antibodies included Anti-GFAP (Z0334, DAKOCytomation, 1:200 IF), β-catenin (610154, BD Transduction Laboratories, 1:75 IF, 1:500 WB), Anti-Par3 (07–330, Millipore, 1:50 IF, 1:500 WB), Anti-Par6 (NBP1-41128 Novus biological, 1:500 WB), Anti-Par6B (sc-166405, Santa Cruz, 1:200) Anti-CD321 (GWB-80389E, GenWay Biotech, 1:100 IF, 1:500 WB), Anti-CD9 (553758, BD Pharmigen, 1:50 IF), Anti-F-actin (ab205, abcam, 1 μg IP), anti-β actin (600-401-886S, Rockland, 1:500 WB), anti-γ tubulin (T5192, Sigma, 1:750 IF), anti-γ actin (A8481, Sigma, 1:500 IF), E-cadherin (3195, Cell Signaling, 1:500 WB), anti-pan actin (4968, Cell Signaling, 1:2,500 WB), Myosin IIa (3403, Cell signaling, 1:50 IF, 1:1,000 WB), Myosin IIb (3404, Cell Signaling, 1:1,000 WB), Anti-ZO-1 (61–7,300, Life technologies, 1:500 WB, 2 μg IP), Anti-ZO1 FITC (339111, Life technologies, 1:100 IF), Occludin-Alexa Fluor 488 (331588, Life Technologies, 1:100 IF), Occludin-Alexa Fluor 594 (331594, Life Technologies, 1:100 IF), Anti-Claudin-3 (34–1,700, Life Technologies, 1:500 WB, 1:150 IF), Occludin (71–1,500, Life Technologies, 1:500 WB), Phalloidin-Alexa Fluor 488 (A12379, Life Technologies, 1:2,000 IF), and Phalloidin-Alexa Fluor 647 (A22287, Life Technologies, 1:80 IF) Other commercial antibodies included normal mouse and rabbit IgG (Santa Cruz), Cy3-conjugated anti-mouse IgG and Cy3-conjugated anti-rabbit (Jackson ImmunoResearch), Alexa Fluor 488-conjugated anti-rabbit, Alexa Fluor 488-conjugated anti-mouse and Alexa Fluor 488-conjugated anti-rat (R37118, R37114, and A-21208, Life Technologies, 1:400) and Latex beads (L1030, Sigma). Reagents: Laminin (L2020, Sigma), Dextran, fluorescein (D1822, ThermoFisher Scientific)

### Magnetic resonance imaging

Mice at postnatal days p8, p10 and at 4 and 44 weeks of age were anaesthetized using Isoflurane (2–3% in O_2_) for the duration of the data acquisition. Ultrasound imaging and MRI were performed at the Animal Imaging Facility at SJCRH. MRI was performed using a 7-Tesla Bruker Clinscan animal MRI scanner (Bruker BioSpin MRI GmbH, Germany) equipped with a Bruker 12S gradient (BGA12S) and a 4-channel phased-array surface coil. Turbo Spin Echo (Fast Spin Echo) protocols (TR 2,500–3,800 ms; TE 39–42 ms) were used to produce T2-weighted images (sagittal, coronal and axial) using a matrix of 320 × 320 and field of view (FOV) of 25 × 25 mm. All images were processed on a Siemens workstation using Syngo MR B15 software (Siemens, Erlangen, Germany).

### Electron microscopy

Electron microscopy was performed at the St Jude Electron Microscopy Facility. Brains from 1-month-old mice were perfused in 4% paraformaldehyde and post-fixed in 2.5% glutaraldehyde in 0.1 M sodium cacodylate buffer. The samples were post-fixed in 2% osmium tetroxide and dehydrated via a graded series of alcohol, cleared in propylene oxide, embedded in epon araldite and polymerized overnight at 70 °C. Sections (70-nm thick) were cut on a Leica Ultracut E. The unstained sections were imaged on a JEOL 1200 EX Transmission Electron Microscope with an AMT 2K digital camera.

### Scanning electron microscopy

One-month-old mice were anaesthetized using Avertin (0.5 mg g^−1^) and perfused with Super reagent perfusion wash and Super reagent perfusion fixation (Electron Microscopy Science, Hatfield, PA). Freshly collected brains were dissected and fixed with 2.5% glutaraldehyde in 0.1 M sodium cacodylate buffer, pH 7.35 (Tousimis Research Corp, Rockville, MD) and 2% osmium tetroxide, pH 7.35, in 0.1 M sodium cacodylate buffer (Electron Microscopy Sciences) before dehydration in an ethanol series and critically point dried (Tousimis Sandai 790, Tousimis Research Corp). Samples were mounted, coated with gold/palladium, and imaged using a JEOL 7000 field emission gun SEM.

### Immunofluorescence and imaging

Brains from 1-month-old mice were perfused with phosphate-buffered 4% paraformaldehyde (PFA) followed by extraction of the brain, embedded in OTC freezing solution and sectioned sequentially from the olfactory bulb lobule towards the cerebellum. Cross-sections were labelled with primary antibodies followed by incubation with Cy3-conjugated secondary antibody (Jackson ImmunoResearch) and Alexa Fluor 488 anti-mouse IgG (Invitrogen). Culture cells were fixed in 3% paraformaldehyde and immunostained with primary antibodies. Cy3 anti-rabbit IgG (Jackson Laboratories) and Alexa Fluor 488 anti-mouse IgG (Invitrogen) were used as secondary antibodies before confocal microscopy imaging. Images were acquired on a Nikon C1si confocal microscope, with a Plan Apo × 40, numerical aperture (NA) 1.3 and/or Plan Apo × 60, NA 1.45 objective (Melville, NY).

### *Ex vivo* microscopy analysis of cilia motility and function

The beating of ependymal cilia was assessed as described previously^44^,^63^,^64^. Briefly, 200-μm vibratome coronal brain slices from 1-month-old animals were placed on a glass chamber with prewarmed Phenol Red-free DMEM/F12. Cilia motility was monitored by differential interference contrast (DIC) on a Nikon C1si confocal microscope, with a Plan Apo × 20, NA 1.45 objective.

To visualize particle movement, vibratome cut brain slices were incubated with prewarmed media (Phenol Red-free DMEM/F12 medium) mixed with a suspension of Latex fluorescent beads (1 μm). The particle flow was monitored by fluorescence microscopy on a Nikon C1si confocal microscope, with a Plan Apo × 60, NA 1.45 objective.

### Histological analysis

For immunohistochemistry, paraffin-embedded brain sections were subjected to deparaffinization and antigen retrieval using standard histology methods. After blocking with 0.1% bovine serum albumin (BSA) and 0.5% Tween 20, the sections were incubated overnight with anti-Alix antibody. The sections were washed and incubated with biotinylated secondary goat anti-rabbit antibody (Jackson ImmunoResearch Laboratory) for 1 h. Endogenous peroxidase was removed by incubating the sections with 0.1% hydrogen peroxide for 30 min. Antibody detection was performed using the ABC Kit and diaminobenzidine substrate (Vector Laboratories, Burlingame, CA), and sections were counterstained with hematoxylin according to standard method. For immunofluorescence, 8-μm cryostat-cut sections were fixed in 3% paraformaldehyde supplemented with 0.1% saponin or 0.2% Triton X-100 for 10 min, followed by blocking with 0.1% BSA, 0.5% Tween 20, 10% normal donkey serum (Jackson Immunoresearch). Subsequently primary antibody (diluted in blocking buffer) was added to the section and incubated overnight. After 1 h of secondary antibody (Jackson Immunoresearch) incubation, sections were washed with blocking buffer w/o normal donkey serum.

### Whole-mount preparations

One-month-old mice were perfused with phosphate-buffered 4% PFA followed by extraction of the brain. The dissected brain was cut in the midline. The overlying cerebral cortex, medial ventricular wall and hippocampus were dissected to reveal the lateral choroid plexus as well as the lateral ventricular wall^65^.

### Tunnel assays

Detection of apoptotic cells in whole-mount preparations of the CP was performed using ApopTag Fluorescein *In Situ* Apoptosis Detection Kit (Millipore), following manufacturer's instructions.

### Evans blue dye injection

The blood–CSF barrier was examined *in vivo* using Evans blue dye, Evans blue dye were administered intraperitoneally (2%, 4 ml kg^−1^ of body weight) and allowed to circulate for 24 h. The animals were then perfused with PBS through the heart and the brain was dissected out. Extravasation of Evans blue dye was visually examined in the ventricular walls of the lateral ventricles by bioluminescence and optical microscopy^66^,^67^,^68^.

### Fluorescein dextran injections

FITC-dextran was injected intraperitoneally in 1-month-old mice (0.7 mg g^−1^ body weight, 10 KDa) and allowed to circulate for 2 h (ref. 69). The brain was then dissected and post-fixed overnight in 4% paraformaldehyde, 0.25% glutaraldehyde in PBS. The CP was isolated as described above and immunostained with ZO-1 antibody. The distribution of FITC-dextran in the CP was visualized by confocal microscopy.

### Generation of CP cell lines with silenced *Alix*

Lentivirus particles were produced by co-transfection of 293T cells with pCAGkGp1R (6 μg), pCAG4-RTR2 (2 μg), pCAG-VSV-G (2 μg) plasmid described by Hanawa *et al*.^70^ and TRC Lentiviral Mouse PDCD6IP (Thermo Scientific) shRNA plasmids (10 μg) or pLKO1 plasmid as mock lentiviral control. Transfections were performed following the manufacturer's instructions (Promega). Immortalized Rat Choroid plexus cells (Z310) were obtained from Dr Zheng of Purdue University^45^. Cells were grown in DMEM medium supplemented with EGF 10 μg ml^−1^ (Peprotech), 10% fetal calf serum, L-glutamine, gentamicin, penicillin and streptomycin. Cells were grown at 37 °C in 5% CO_2_. A total of 0.5 × 10^6^ Z31O cells were seeded into 10-cm plates. After 24 h, cells were transduced with empty vector or *Alix* shRNA lentiviral particles in a total volume of 3 ml in the presence of 8 μg ml^−1^ polybrene (Chemicon). After 72 h, infected cells were selected with 2 μg ml^−1^ puromycin for 5 days. All cell lines were checked and confirmed negative for myoplasma.

### Epithelial permeability assay

Z310 *Alix* shRNA no. 2 and control cells (2.5 × 10^4^) were cultured in supported permeable membranes (Corning) coated with laminin. After 48 h FITC-dextran (0.5 mg ml^−1^) was added to the apical or basal chamber and incubated for additional 3 h. The flux of FITC-labelled dextran (excitation, 485–12 nm; emission, 520 nm) across the cell monolayer into the basal or apical medium was determined using a BMG Omega plate reader. For confocal analysis of the fluorescent tracer, cells were washed twice with PBS and fixed as described above.

### Actomyosin purification

Actomyosin was purified according to the procedure of Larson *et al*.^71^. In brief, whole brain was rapidly removed and place in PBS 0 °C. Meninges and cerebellum were removed by dissection and the tissue was place in a 2-ml Dounce tissue grinder containing cold homogenization buffer (10 mM Tris-HCl (pH 7.5), 100 mM KCl, 1.0 mM EDTA pH 8.0 and 0.2 mM 2-mercaptoethanol). The sample was homogenized with 15 total strokes of a large clearance pestle. The homogenate was kept on ice with slight agitation for 90 min and centrifuged at 23,500 *g* for 1 h. The resulting supernatant (S1) was dialysed overnight against two changes of the same buffer. The fine precipitate was collected by centrifugation at 10,000 *g* for 15 min. The pellet was resuspended in buffer (50 mM Tris-HCl (pH 7.5), 10 mM KCI, 1% Triton X-100, 0.2 mM 2-mercaptoethanol) and centrifuged at 10,000 *g* for 5 min. The extraction was repeated twice and followed by washings with the same buffer w/o Triton X-100. The pellet was solubilized in 50 mM Tris-HCl (pH 7.5) supplemented with 800 mM KCl and incubated overnight on ice. An equal volume of glycerol was added. The brain actomyosin was stored at −20 °C.

### Determination of ATPase activities

After actomyosin purification, the ATPase activity was determined by measuring the inorganic phosphate released from ATP^71^,^72^. The reactions, carried out at 37 °C in a total volume of 0.2 ml were initiated by addition of 1.0 mM ATP, final concentration. The measurement of the K^+^-ATPase activity was carried out in 25 mM imidazole-HCl buffer (pH 8.0), 0.5 mM EDTA, 0.1 mM dithiothreitol (DTT) and 600 mM KCl; the Mg^2+^-ATPase activity, in 25 mM imidazole-HCl buffer (pH 7.0), 2.5 mM MgCl_2_, 0.1 mM DTT and 50 mM KCl; the Ca^2+^-ATPase activity, in 25 mM imidazole-HCl buffer (pH 7.0), 10 mM CaCl_2_, 0.1 mM DTT and 50 mM KCI. One to 2 μg actomyosin protein was used per reaction. For 80 μl test solution containing inorganic phosphate (standard final concentrations: 2.5, 5.0, 10.0, 20.0, 40.0, 80.0 and 160.0 μmol), 60 μl Triton X- 100 was added and then 60 μl ammonium molybdate reagent. After standing for 10 min, the absorbance of the test solution was read at 375 nm against a blank.

### Subcellular fractionation from brain tissue

Subcellular fractionation protocol was adapted from Hallett P. *et al*.^73^. In brief, a mouse brain was lysed using ice-cold lysis buffer (10 mM Tris-HCl (pH 7.4), 320 mM sucrose, 10 mM Tris base, 5 mM NaF, 1 mM Na_3_VO_4_, 1 mM EDTA, 1 mM EGTA, protease inhibitors) by using a Dounce homogenization (15 strokes). Homogenates were centrifuged for 10 min at 800 *g* at 4 °C. The resulting supernatant (S1) was centrifuged for 2 h at 165,000 *g* at 4 °C. The consequent supernatant (S2) was transfered into a clean tube and the pellet (P2) fraction was solubilized in ice-cold resuspension buffer (10 mM Tris-HCl (pH 7.4). 1% sodium deoxycholate, 5 mM NaF, 1 mM Na_3_VO_4_, 1 mM EDTA, 1 mM EGTA, protease inhibitors). The lysates were subjected to BCA Protein Assay (Thermo Scientific) to measure protein concentrations.

### Co-immunoprecipitations

Seventy microgram brain subcellular fraction P2 lysates were incubated with 2 μg of anti-ZO-1 Par3, and Par6 antibodies or 10 μg of anti-F-actin antibody overnight at 4 °C. Samples were immunoprecipitated with PureProteome Protein A/G Mix Magnetic Beads coupled to V5 peptide for 1 h at 4 °C. The beads were washed three times with ice-cold resuspension buffer (10 mM Tris-HCl (pH 7.4), 1% sodium deoxycholate, 10 mM Tris base, 5 mM NaF, 1 mM Na_3_VO_4_, 1 mM EDTA, 1 mM EGTA, protease inhibitors), and one time with resuspension buffer without detergent. Bound proteins were released by boiling the beads with sample buffer and run on SDS polyacrylamide gels under denaturing conditions followed by immunoblotting with the indicated antibodies.

### Western blotting

Protein concentration of actomyosin was determined at OD 595, using BSA as standard. 2.0 μg of purified actomyosin were electrophoresed on NuPAGE 4–12% gels (Invitrogen) and wet-blotted to PVDF membranes overnight at 30 mA. Membranes were probed with specific antibodies at the dilutions listed above, followed by HRP-conjugated goat anti-rabbit or anti-mouse IgG (Jackson ImmunoResearch Laboratories). Signals were detected with a WestFemto maximum sensitivity substrate kit (Thermo Scientific). Immunoblots were photographed using a BioRad Chemidoc PM Molecular Imager and band densities were measured using ImageJ64 software. Montages were assembled using Adobe Illustrator, and then converted to TIFF files. Full gel scans can be found in Supplementary Figs 11–13.

### Culture of epithelial cells

Mouse trachea epithelial cells cultures were established and cultured as previously described by You Y. *et al*.^74^. In brief, kill mouse in CO_2_ chamber and the trachea (WT: *n*=10, *Alix*^*−/−*^: *n*=11) was removed under sterile conditions and washed with sterile Ham's F12 medium. The tracheas were incubated in pronase solution overnight at 4 °C. Warm fetal bovine serum (FBS) was added to a final concentration of 10% (v/v) and samples were inverted gently 15–20 times to detach epithelial cells from the trachea. The digested solution containing the enzyme released cells were centrifuged at 500 *g* for 10 min. Supernatant was aspirated. Cells were resuspended in DNase solution, incubated on ice for 5 min, and were centrifuged at 500 *g* for 5 min. Cells were resuspended in mTEC basic medium (DMEM/Ham's F-12 (DMEM/F-12) supplemented with 15 mM HEPES, 4 mM L-Glutamine, 3.6 mM NaHCO_3_, 100 U ml^−1^ penicillin, and 100 mg ml^−1^ streptomycin) containing 10% FBS and plated in a culture dish. Cells were incubated at 37 °C, 5% CO_2_ for 3–4 h allowing fibroblasts to attach. These floating tracheal cells were collected by centrifuging at 500 *g* for 5 min and grown in mTEC/Plus medium (mTEC/Basic medium supplemented with 10 mg ml^−1^ insulin, 5 mg ml^−1^ transferrin, 0.1 mg ml^−1^ CT, 25 ng ml^−1^ EGF, 30 mg ml^−1^ BPE, 5% FBS (v/v) and 0.015 μg ml^−1^ retinoic acid) for immunofluorescence or SEM analysis.

### EGTA treatment

Z310 cells (0.4 × 10^5^) were seeded onto glass coverslips treated with poly-D-Lysine and coated with 10 μg cm^−2^ laminin. Forty eight hours after seeding, cells were replaced with 1 ml of medium containing 2 mM of EGTA; control cells were replaced with fresh medium without EGTA and incubated at 37 °C in 5% CO_2_. After 20 min, the EGTA medium was replaced with complete medium and cell were incubated for 1 h at 37 °C in 5% CO_2_ to reconstitute cell–cell contacts.

### Cytochalasin D treatment

CD was added to the culture medium at final concentrations of 10 μM and cells were incubated for 1 h at 37 °C in 5% CO_2_. The CD medium was replaced with complete medium and cells were incubated for 1 h at 37 °C in 5% CO_2_ to reconstitute cell–cell contacts.

### Statistical analysis

Data were expressed as mean±s.d. and evaluated using Student's *t*-test for comparison with WT samples. Mean differences were considered statistically significant when *P* values were <0.05 (*).

### Data availability

All the data needed to evaluate the results and conclusions of the experimental work are included in the paper and/or the Supplementary Materials, and fully available. Additional data or information related to this paper may be requested from the authors.

## Additional information

**How to cite this article:** Campos, Y. *et al*. Alix-mediated assembly of the actomyosin–tight junction polarity complex preserves epithelial polarity and epithelial barrier. *Nat. Commun.* 7:11876 doi: 10.1038/ncomms11876 (2016).

## Supplementary Material

Supplementary Figures Supplementary Figures 1-13

Supplementary Movie 1 3D reconstructed whole-mount scanning electron microscopy (SEM) images showed the organization of the lateral CP in WT.

Supplementary Movie 2 3D reconstructed whole-mount scanning electron microscopy (SEM) images showed the organization of the lateral CP in Alix^-/-^ mice.

Supplementary Movie 3 3D reconstruction of scanning laser confocal microscopy images of WT choroid plexus stained for CD9.

Supplementary Movie 4 3D reconstruction of scanning laser confocal microscopy images of Alix^-/-^ choroid plexus stained for CD9.

Supplementary Movie 5 3D reconstruction of whole-mount scanning electron microscopy (SEM) images showed the organization and orientation of cilia of the lateral wall in WT control.

Supplementary Movie 6 3D reconstruction of whole-mount scanning electron microscopy (SEM) images showed the organization and orientation of cilia of the lateral wall in Alix^-/-^. The Alix^-/-^ cilia are of different sizes and are also highly disorganized.

Supplementary Movie 7 Cilia beating of ependymal cells from WT mice. Micro-video recording of lateral wall shows the beating motion of cilia in WT control.

Supplementary Movie 8 Cilia beating of ependymal cells from Alix^-/-^ mice. The Alix^-/-^ motile cilia display an asynchronous beating.

Supplementary Movie 9 Micro-video showing the ependymal flow in WT lateral wall.

Supplementary Movie 10 The speed of ependymal flow was significantly reduced in Alix^-/-^ mice. The speeds of fluorescent beads on the brain coronal section preparation were slower and stagnate in Alix^-/-^ mice compare to WT control.

Supplementary Movie 11 3D-Reconstructed volume of whole-mount confocal microscopy images of WT choroid plexus stained with phalloidin.

Supplementary Movie 12 3D-Reconstructed volume of whole-mount confocal microscopy images of Alix^-/-^ choroid plexus stained with phalloidin showed the organization of the CP compared to WT.

Supplementary Movie 13 3D-confocal reconstruction of WT whole-mount CP showing the expression pattern of ZO-1

Supplementary Movie 14 3D-confocal reconstruction of Alix^-/-^ whole-mount CP showing the expression pattern of ZO-1

Supplementary Movie 15 3-D reconstruction of scanning laser confocal microscopy Z-stacks of WT choroid plexus immunostained for Occludin1.

Supplementary Movie 16 3-D reconstruction of scanning laser confocal microscopy Z-stacks of Alix^-/-^ choroid plexus immunostained for Occludin1.
