# Supplementary material for: Alix-mediated assembly of the actomyosin–tight junction polarity complex preserves epithelial polarity and epithelial barrier
Source: Nat Commun. 2016 Jun 23;7:11876. doi: 10.1038/ncomms11876 (PMC4931029; doi:10.1038/ncomms11876)
Supplement: Supplementary Figures — 1-13 [file ncomms11876-s1.pdf]

SUPPLEMENTARY INFORMATION

Supplementary Figure 1

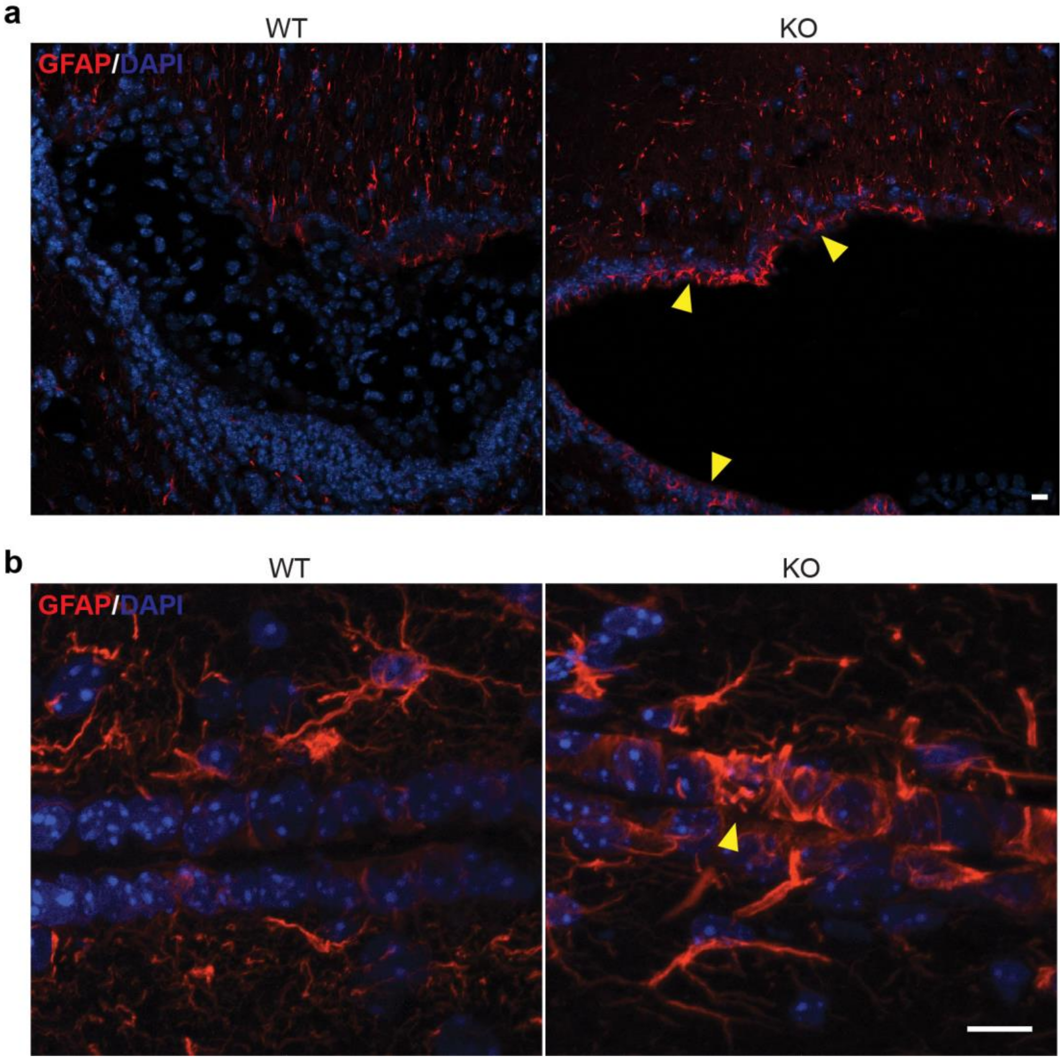

Supplementary Figure 1 (**a**) GFAP immunofluorescence (red) of WT and *Alix−/−* lateral walls. Scale bar, 10 μm. (**b**) Higher magnification of (**a**) showing GFAP (red) immunostaining of astrocytes covering the ependymal layer of the *Alix−/−* ventricular surface. Scale bar, 100 nm. Nuclei were stained with DAPI (blue).

Supplementary Figure 2

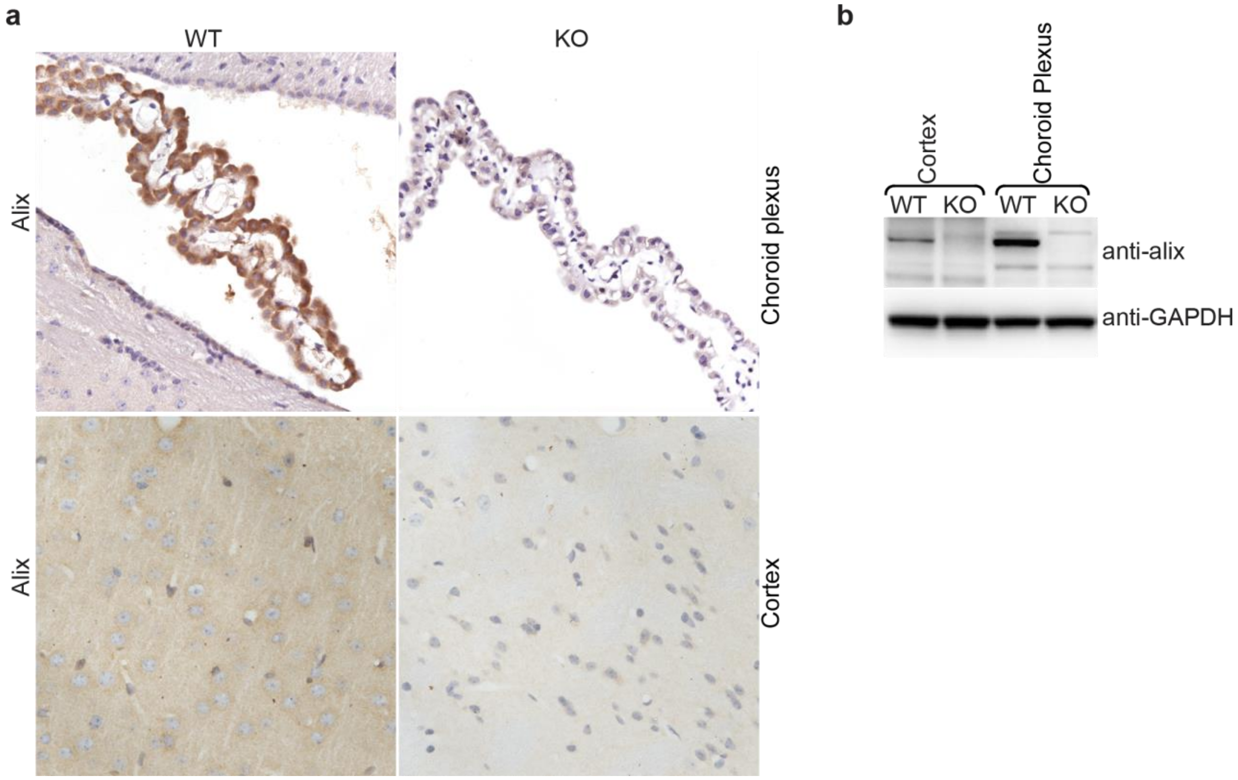

Supplementary Figure 2 (**a**) Immunohistochemical staining of coronal paraffin sections of the WT brain showed high Alix expression in CP and overall low expression in cerebral cortex. (**b**) Western blot analysis confirmed the relative levels of Alix protein in total lysates from WT cortex and CP. The specificity of Alix antibody was validated by lack of positive signal or band in knockout sample.

Supplementary Figure 3

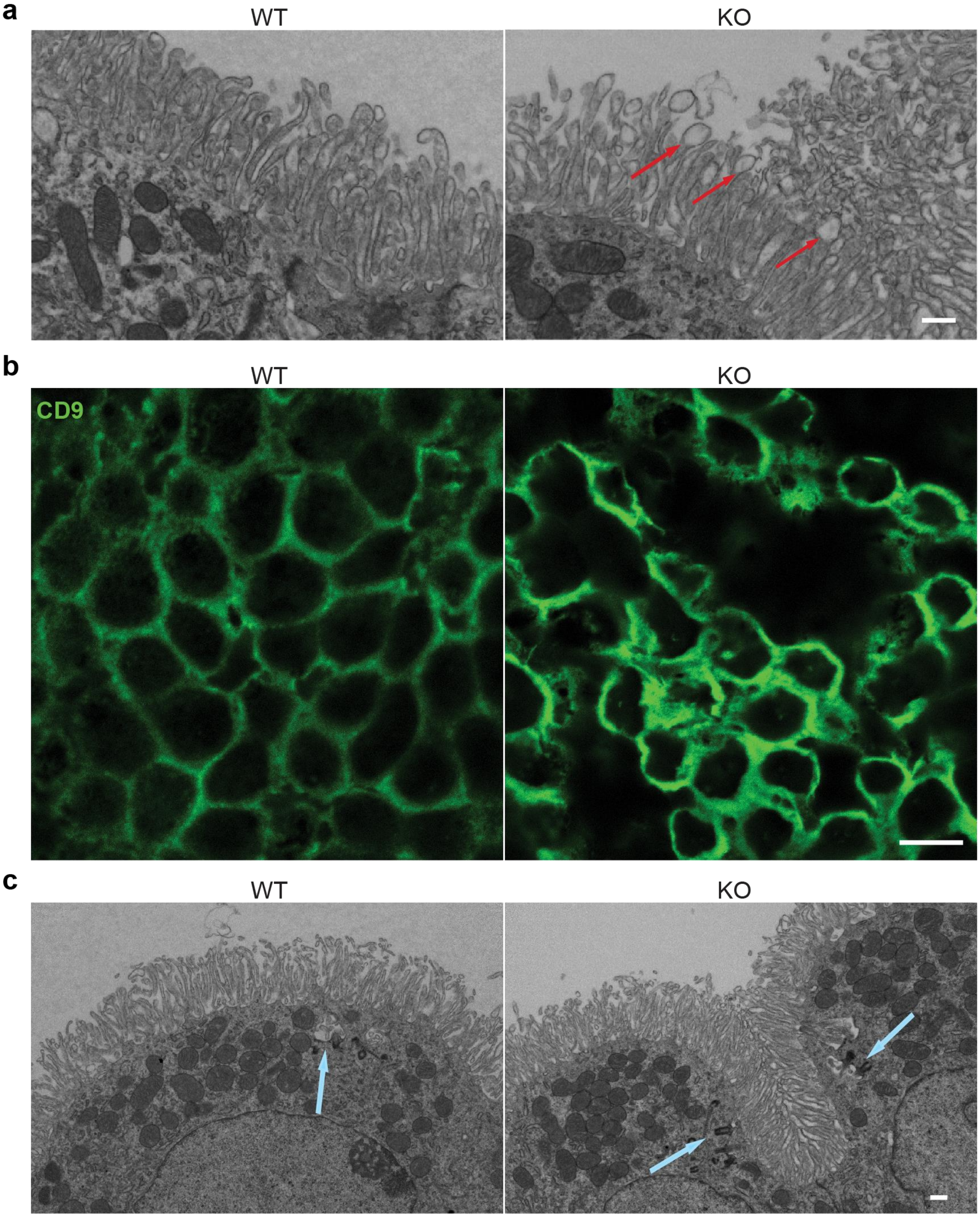

Supplementary Figure 3 (**a**) Transmission electron microscopy (TEM) micrographs of cross-sections of the WT and *Alix*$^{-/-}$ CP. Arrows point to the abnormal blebbing of

microvilli in the mutant CP. Scale bar, 0.5 μm. (**b**) Whole-mount WT and *Alix*$^{-/-}$ CP immunostained for CD9, a marker of the microvilli. Scale bar, 10 μm. (**c**) TEM micrographs of *Alix*$^{-/-}$ CP showing the mislocalization of the basal bodies in the mutant CP. Scale bar, 500 nm.

Supplementary Figure 4

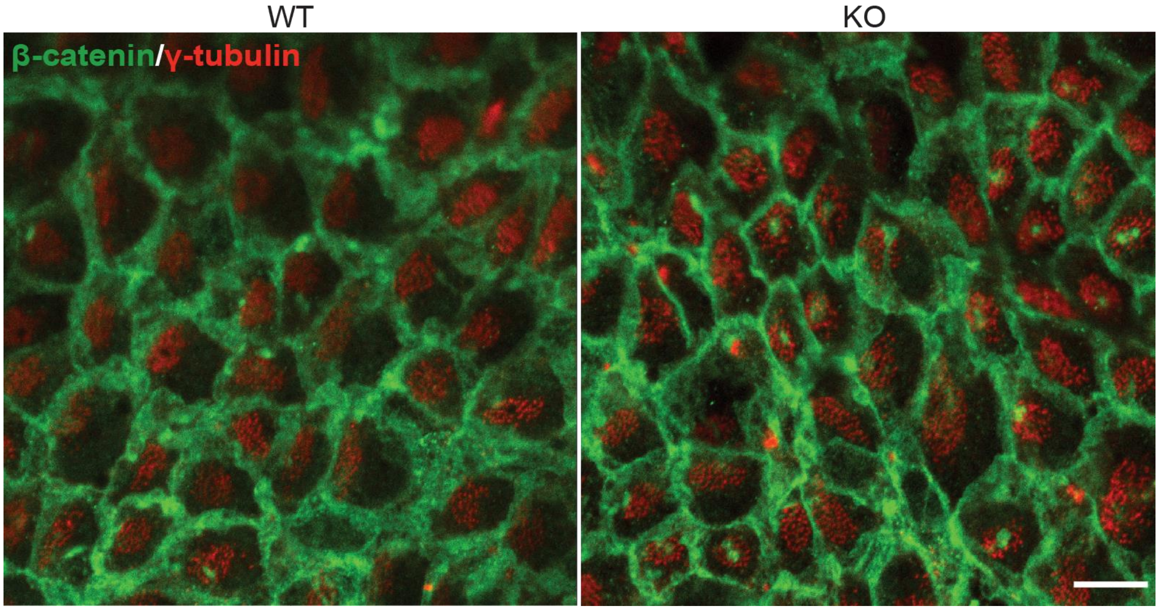

Supplementary Figure 4 Abnormal sizes of ependymal cells and positioning of the basal bodies in the *Alix*$^{-/-}$ lateral wall. Whole-mount preparations of the WT and *Alix*$^{-/-}$ lateral walls immunostained for β-catenin (green) and γ-tubulin (red). Scale bar, 10 μm.

Supplementary Figure 5

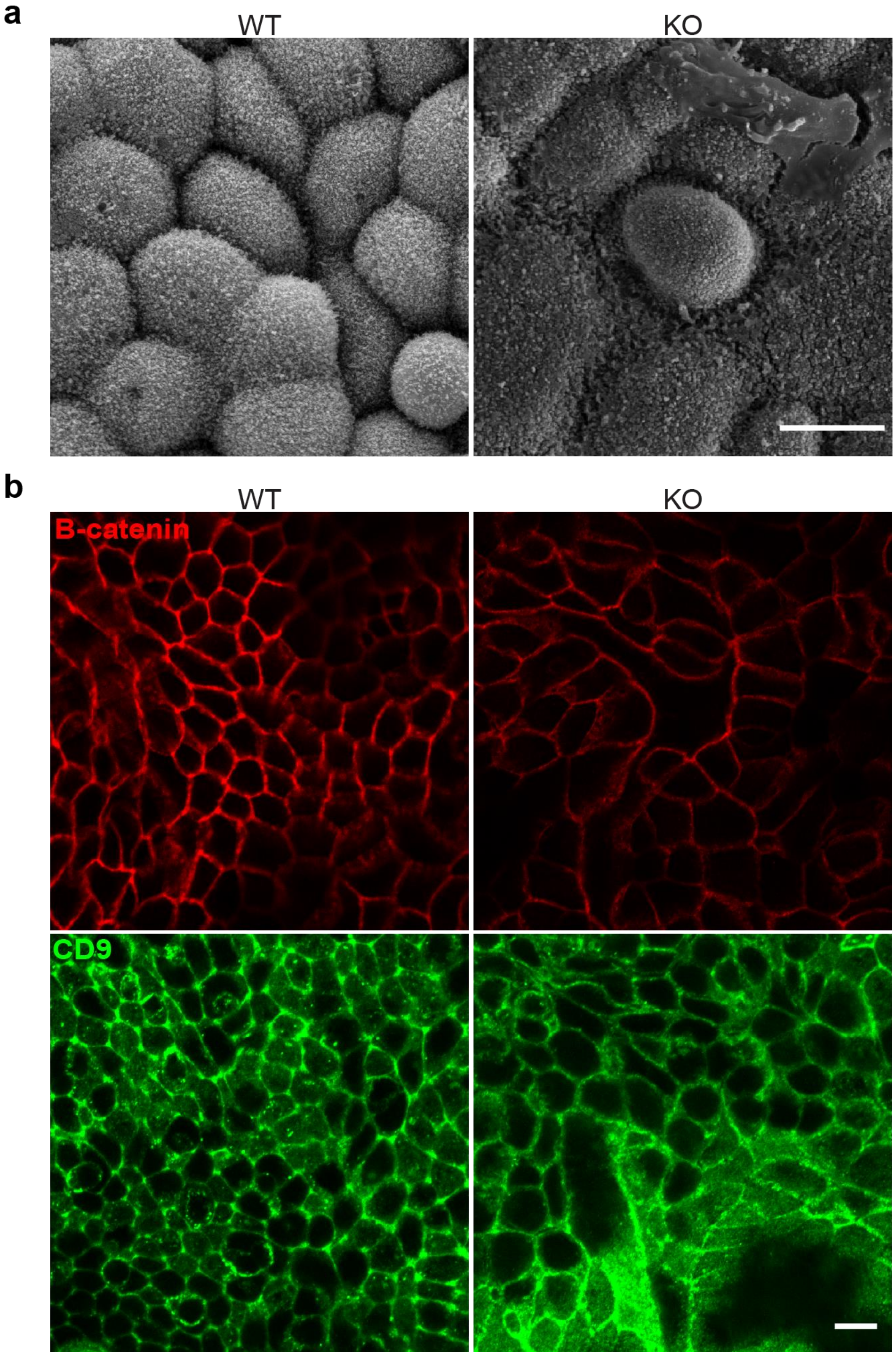

Supplementary Figure 5 Apoptotic cell extrusion in *Alix*$^{-/-}$ epithelia**.** (**a**) SEM images of WT and *Alix*$^{-/-}$ CP. Scale bar, 10 μm**.** (**b**) Tracheal epithelia cells isolated from WT and *Alix*$^{-/-}$ mice and immunostained for β-catenin (red) and CD9 (green). Scale bar, 10 μm.

Supplementary Figure 6

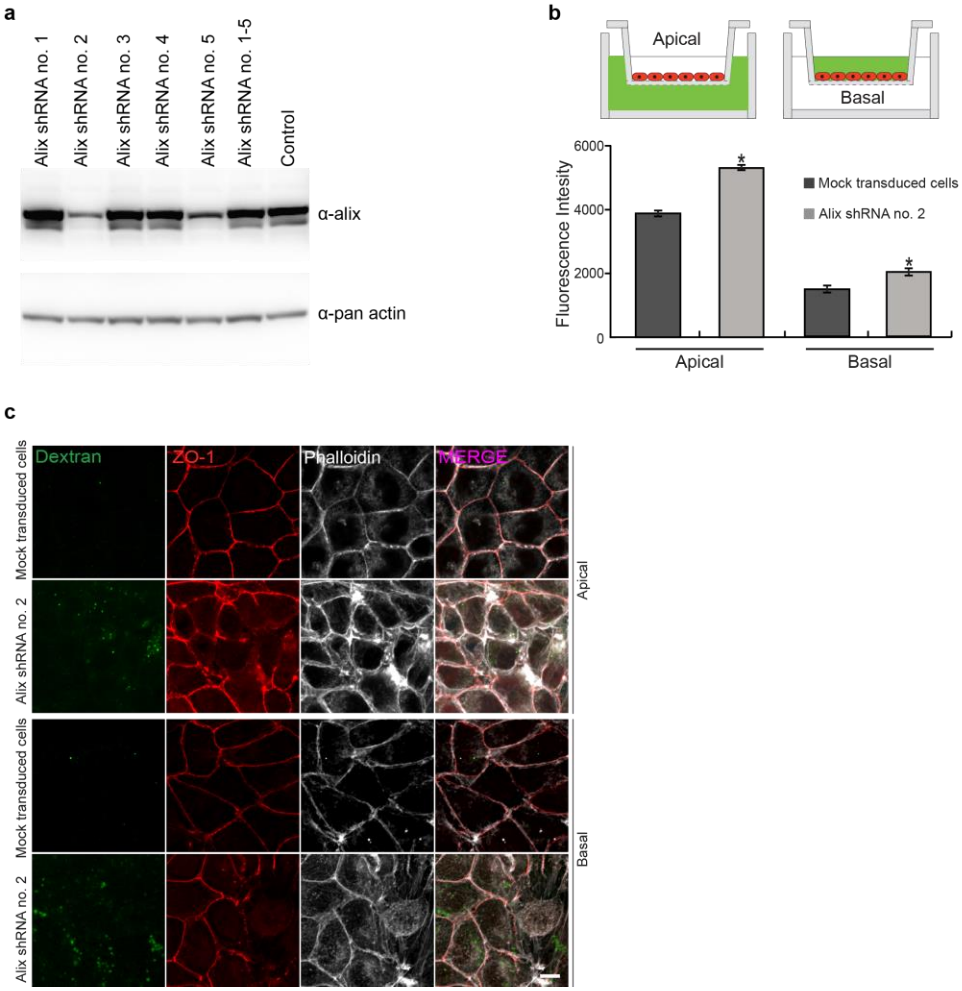

Supplementary Figure 6 *Alix* knockdown in Z310 choroid plexus cells affect the flux of FITC dextran. **(a)** Western blot analysis of Alix in lysates of Z310 CP cells transduced with lentiviral vectors expressing control shRNA or *Alix* shRNA no. 1 - no. 5. Note that shRNA no. 2 knocks down Alix most efficiently. **(b)** Schematic representation of the supported permeable membrane culture and quantification of the fluorescence recovered in the apical or basal chamber after addition of FITC dextran in the opposite chambers. **(c) F**luorescence imaging of FITC-dextran (green) in Z310 cells with WT or silenced Alix expression immunostained for ZO-1 (red) and Phalloidin (white). Scale bar, 10 μm.

Supplementary Figure 7

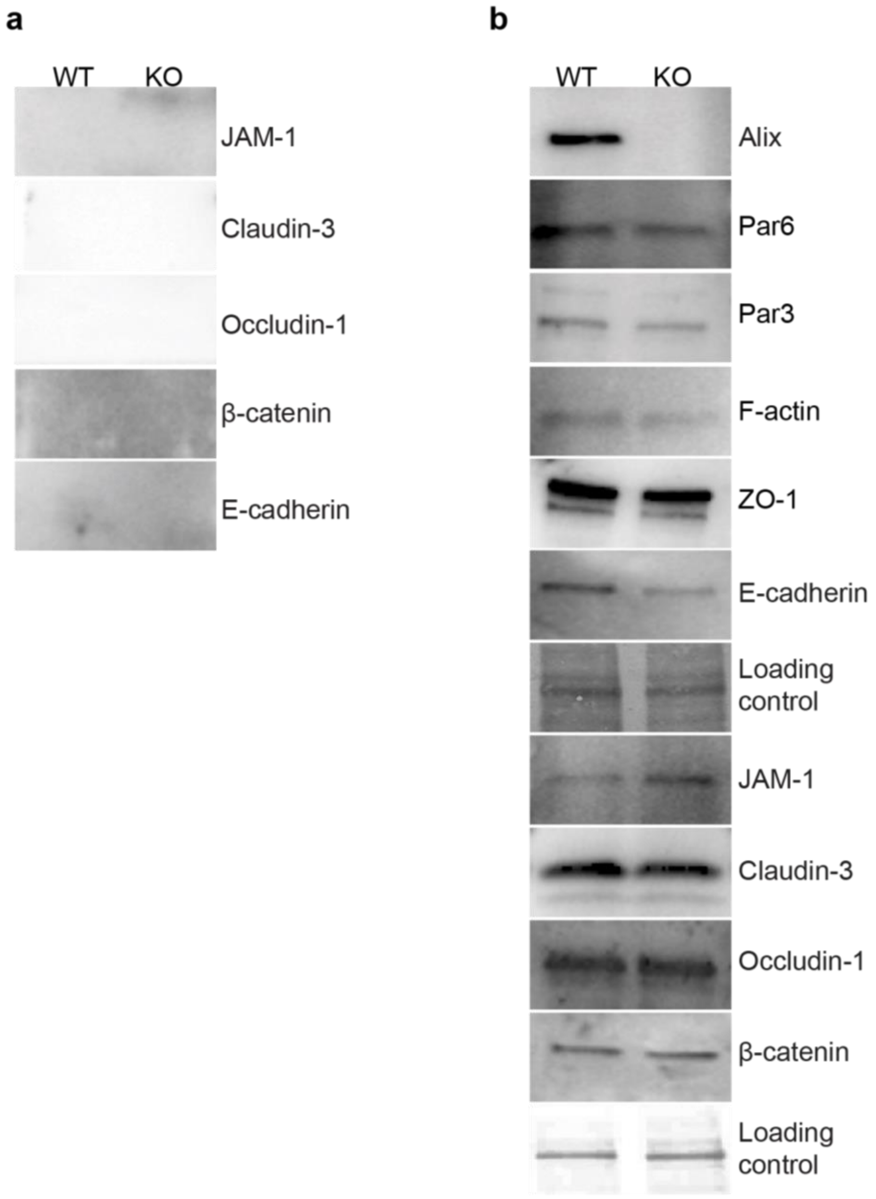

Supplementary Figure 7 (a) Immunoblots of purified actomyosin preparations probed with antibodies agains JAM-1, Claudin-3, Occludin, β-catenin and E-cadherin. **(b)** Immunoblots of WT and *Alix*$^{-/-}$ membranous/cytoskeletal subcellular fractions probed with antibodies against the indicated proteins prior to immunoprecipitation.

Supplementary Figure 8

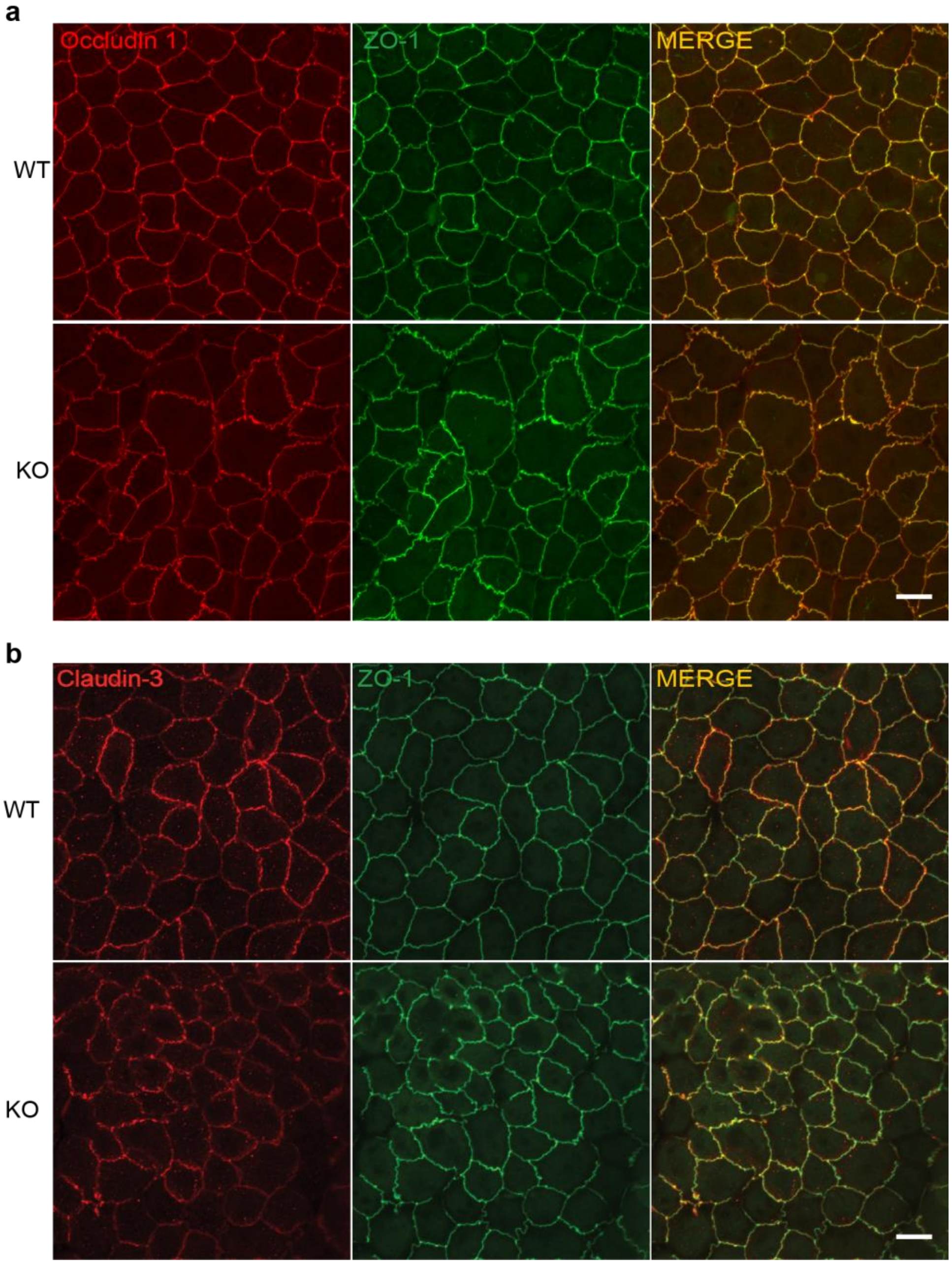

Supplementary Figure 8 Abnormal organization of the tight junction in *Alix*$^{-/-}$ CP. (**a**) Confocal images of whole-mount WT and *Alix*$^{-/-}$ CP immunostained for Occludin (red) and ZO-1 (green). (**b**) Confocal images of whole-mount CP immunostained for Claudin 3 (red) and ZO-1 (green). Scale bars, 10 μm.

Supplementary Figure 9

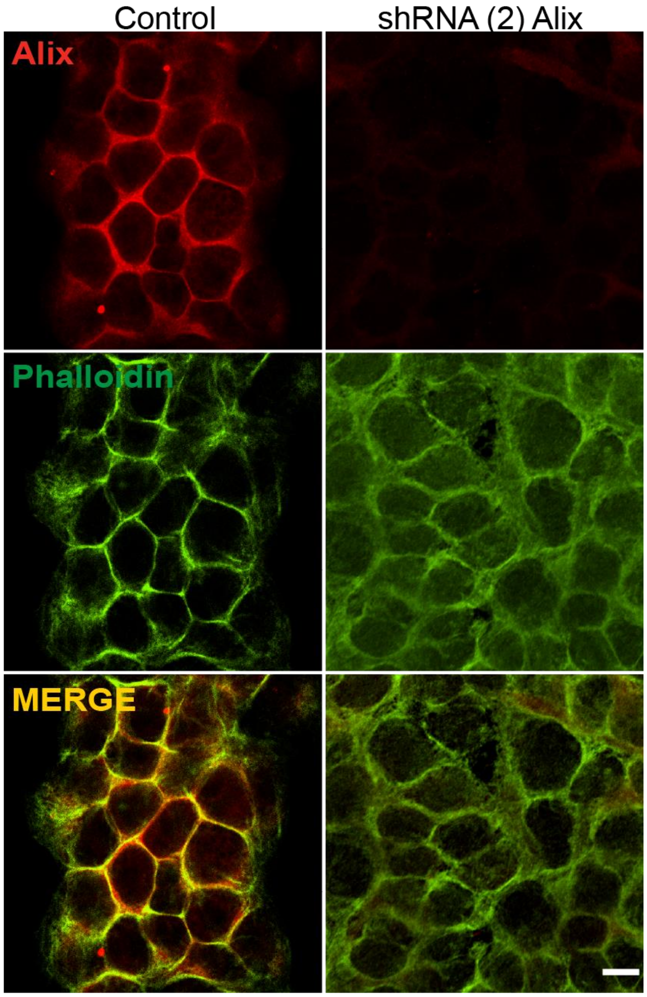

Supplementary Figure 9 Alix (red) and phalloidin (green) immunofluorescent labeling of Z310 cells transduced with control shRNA lentivirus or with *Alix* shRNA no. 2. Scale bar, 10 μm.

Supplementary Figure 10

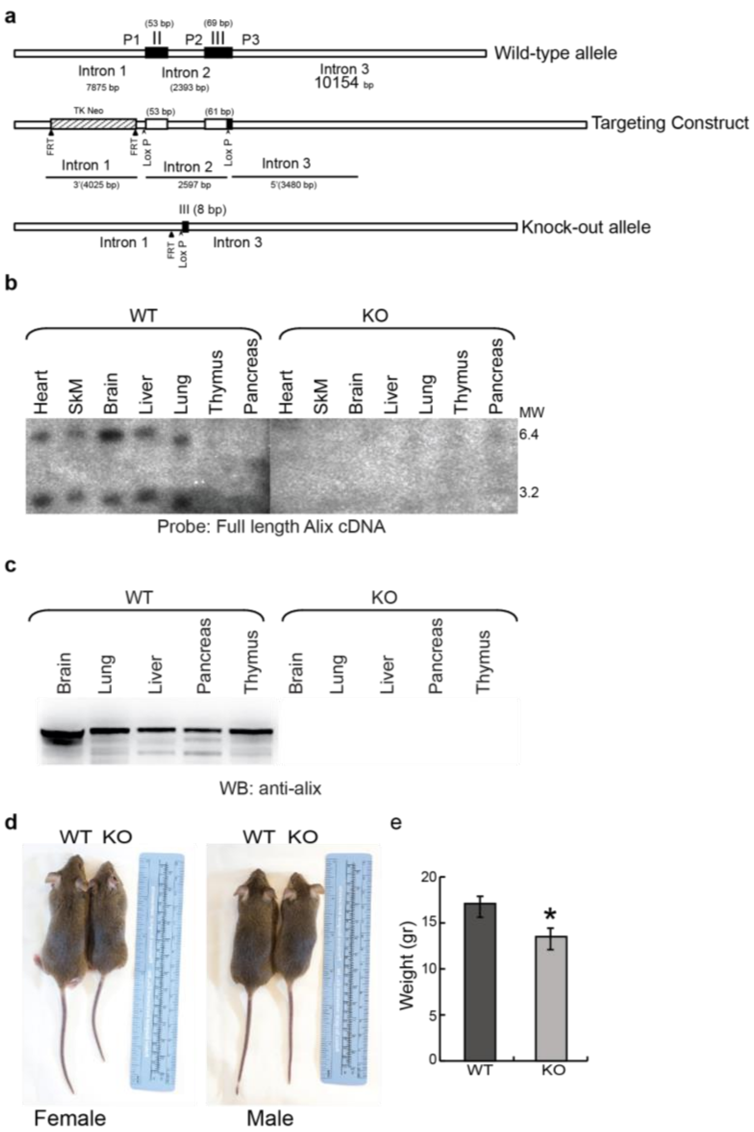

Supplementary Figure 10 Generation of *Alix*$^{-/-}$ mice. (**a**) Schematic representation of the Alix targeting construct**.** (**b**) Northern blot analysis of Alix expression in WT and *Alix*$^{-/-}$ heart, skeletal muscle, brain, liver, lung, thymus, and pancreas from 1-month-old mice. (**c**) Western blot analysis with anti-Alix antibody revealed absence of the Alix protein in lysates of brain, lung, liver, pancreas, and thymus from *Alix*$^{-/-}$ mice. (**d**) Comparison of the body size of *Alix*$^{-/-}$ with the WT counterparts. (**e**) Quantification of the weight of WT and *Alix*$^{-/-}$ mice. Data represent the mean ±SD (n=10).

Supplementary Figure 11

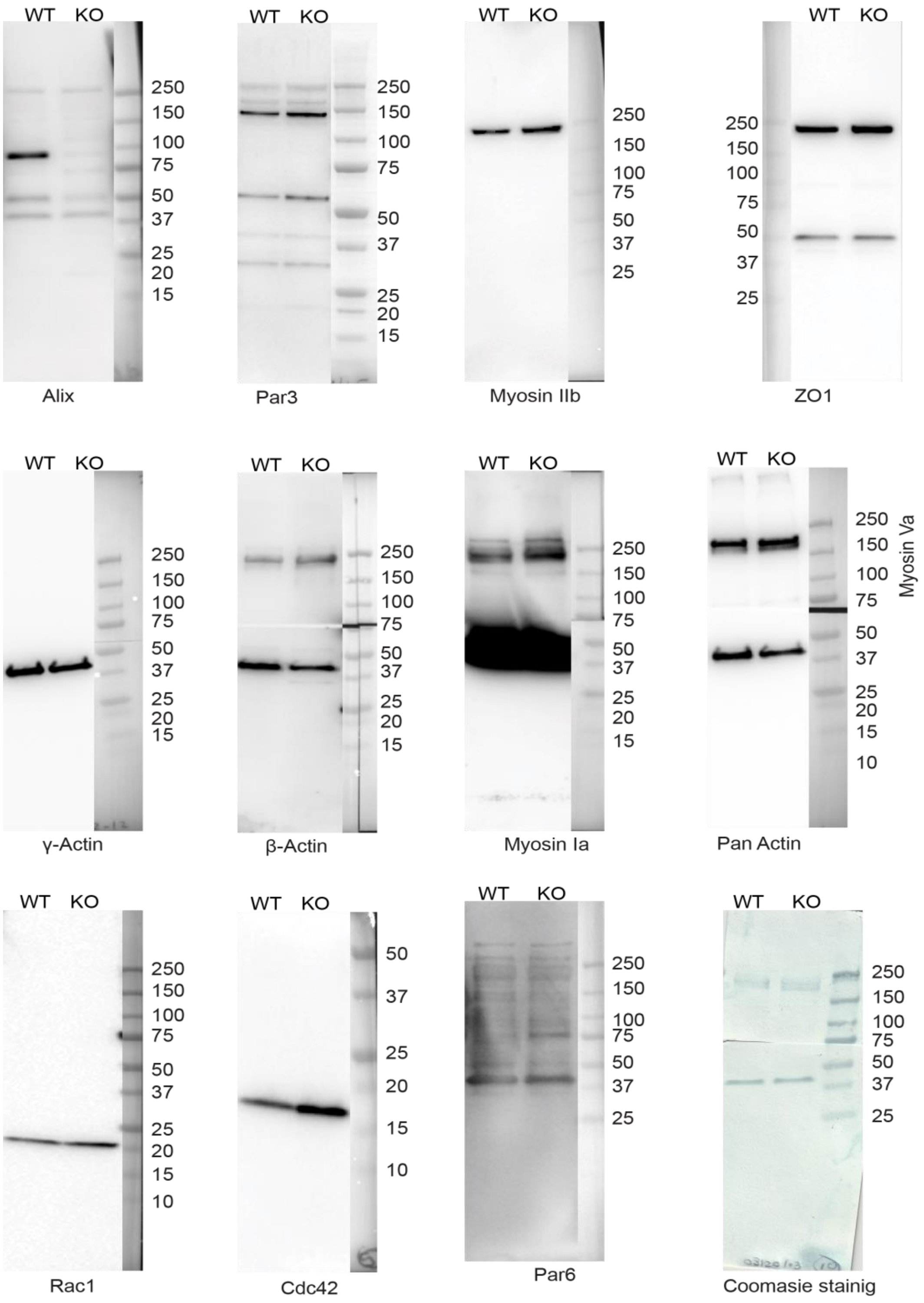

Supplementary Figure 11. Whole unaltered Western blots from Figure 6d. Labeling is consistent with Figure 6d.

Supplementary Figure 12

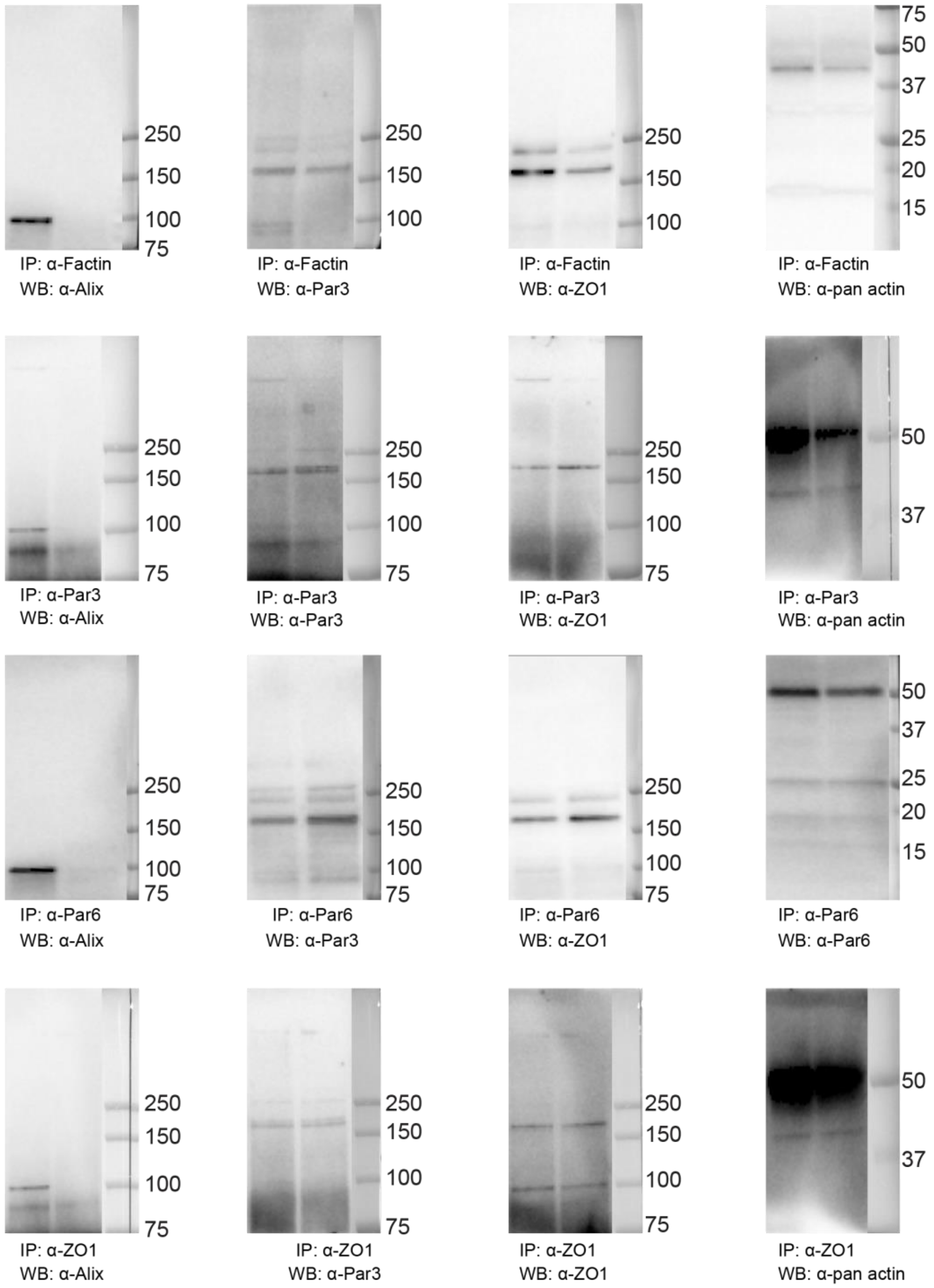

Supplementary Figure 12. Whole unaltered Western blots from Figure 6f. Labeling is consistent with Figure 6f.

Supplementary Figure 13

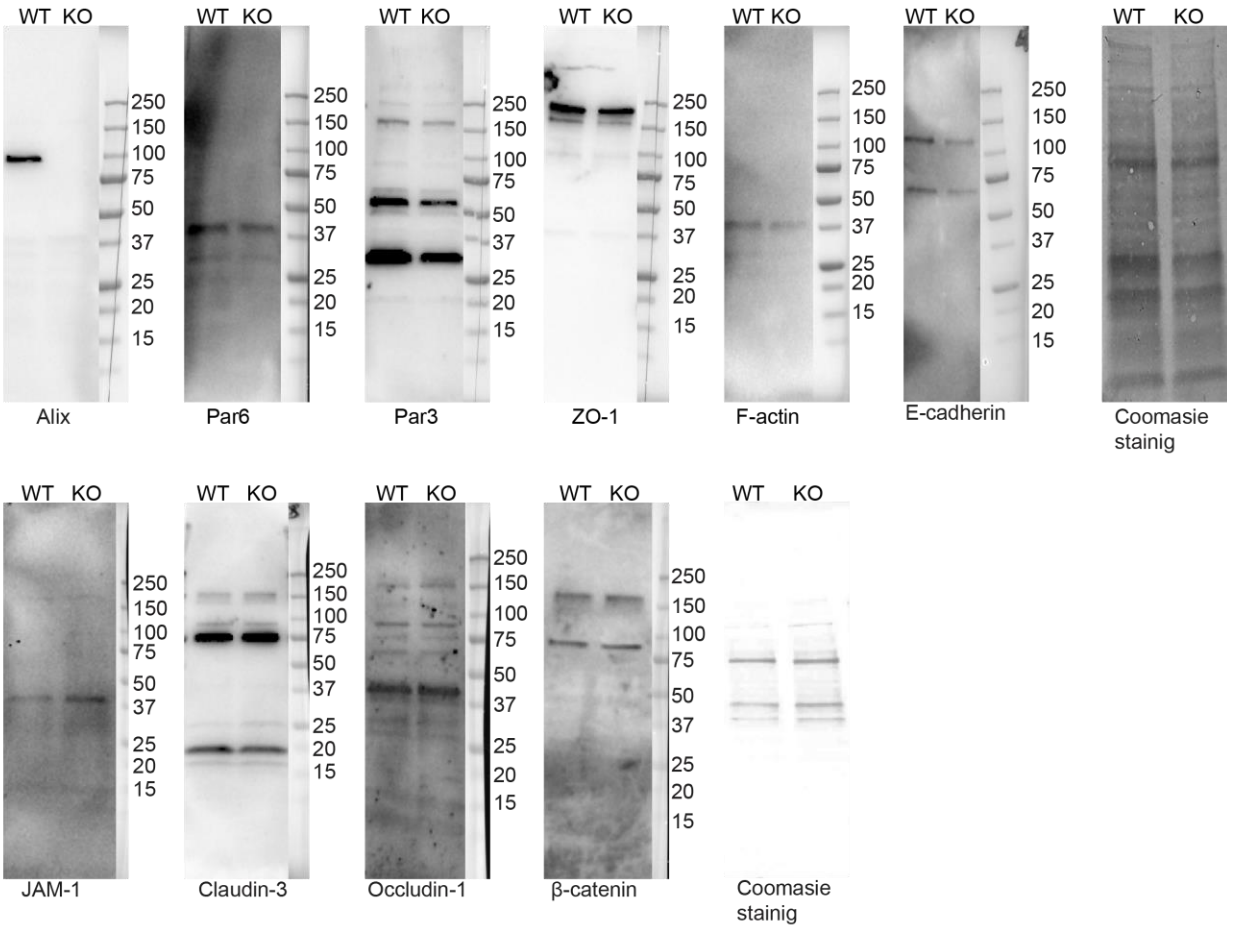

Supplementary Figure 13. Whole unaltered Western blots from Supplementary Fig. 7b. Labeling is consistent with Supplementary Fig. 7b.
